# FGF23 directly inhibits osteoprogenitor differentiation in *Dmp1*-knockout mice

**DOI:** 10.1172/jci.insight.156850

**Published:** 2023-12-22

**Authors:** Guillaume Courbon, Dominik Kentrup, Jane Joy Thomas, Xueyan Wang, Hao-Hsuan Tsai, Jadeah Spindler, John Von Drasek, Laura Mazudie Ndjonko, Marta Martinez-Calle, Sana Lynch, Lauriane Hivert, Xiaofang Wang, Wenhan Chang, Jian Q. Feng, Valentin David, Aline Martin

**Affiliations:** 1 Division of Nephrology and Hypertension, Center for Translational Metabolism and Health, Feinberg School of Medicine, Northwestern University, Chicago, Illinois, USA.; 2 Texas A&M School of Dentistry, Texas A&M University, Dallas, Texas, USA.; 3 Department of Medicine, University of California, San Francisco, San Francisco, California, USA.; 4 Shanxi Medical University School and Hospital of Stomatology, Clinical Medical Research Center of Oral Diseases of Shanxi Province, Taiyuan, China.

**Keywords:** Bone Biology, Metabolism, Bone disease, Genetic diseases, Osteoclast/osteoblast biology

## Abstract

Fibroblast growth factor 23 (FGF23) is a phosphate-regulating (Pi-regulating) hormone produced by bone. Hereditary hypophosphatemic disorders are associated with FGF23 excess, impaired skeletal growth, and osteomalacia. Blocking FGF23 became an effective therapeutic strategy in X-linked hypophosphatemia, but testing remains limited in autosomal recessive hypophosphatemic rickets (ARHR). This study investigates the effects of Pi repletion and bone-specific deletion of *Fgf23* on bone and mineral metabolism in the dentin matrix protein 1–knockout (*Dmp1*^KO^) mouse model of ARHR. At 12 weeks, *Dmp1*^KO^ mice showed increased serum FGF23 and parathyroid hormone levels, hypophosphatemia, impaired growth, rickets, and osteomalacia. Six weeks of dietary Pi supplementation exacerbated FGF23 production, hyperparathyroidism, renal Pi excretion, and osteomalacia. In contrast, osteocyte-specific deletion of *Fgf23* resulted in a partial correction of FGF23 excess, which was sufficient to fully restore serum Pi levels but only partially corrected the bone phenotype. In vitro, we show that FGF23 directly impaired osteoprogenitors’ differentiation and that DMP1 deficiency contributed to impaired mineralization independent of FGF23 or Pi levels. In conclusion, FGF23-induced hypophosphatemia is only partially responsible for the bone defects observed in *Dmp1*^KO^ mice. Our data suggest that combined DMP1 repletion and FGF23 blockade could effectively correct ARHR-associated mineral and bone disorders.

## Graphical abstract

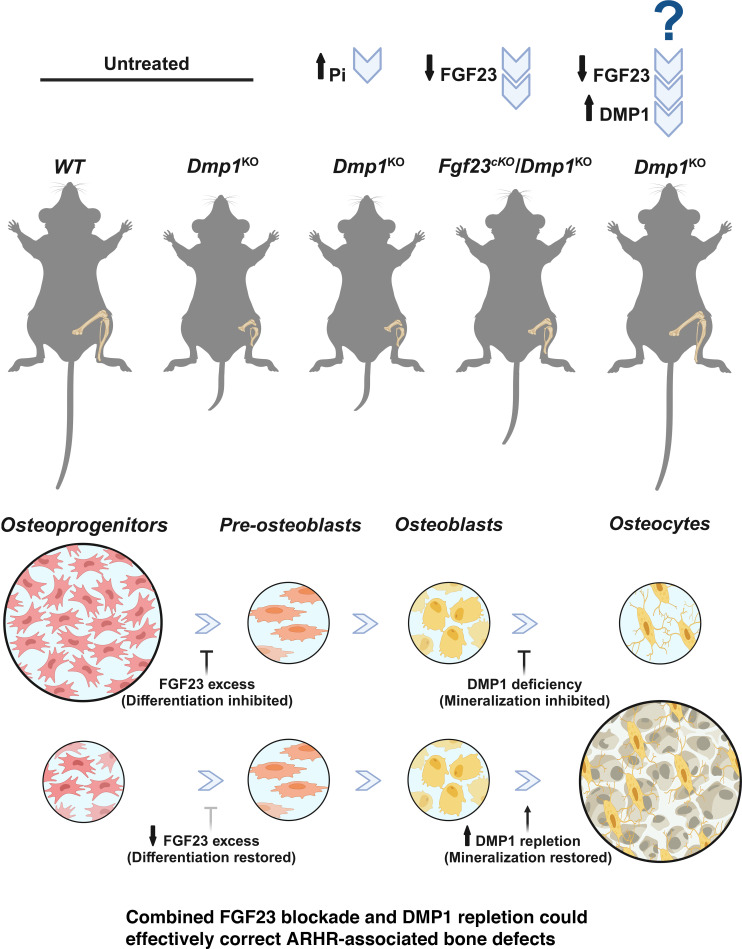

## Introduction

Fibroblast growth factor 23 (FGF23) is a phosphate (Pi) and vitamin D–regulating hormone produced by osteocytes (1, 2). In hereditary hypophosphatemic disorders, such as X-linked hypophosphatemia (XLH) and autosomal recessive hypophosphatemic rickets (ARHR), the primary increase in circulating FGF23 levels leads to renal Pi wasting, causing hypophosphatemia and secondary alterations in mineral and bone metabolism, such as hyperparathyroidism, rickets, and osteomalacia (3–7). XLH and ARHR are rare but debilitating disorders that lead to bone deformities, short stature, handicap, and devastating rheumatologic and metabolic complications. To date, the most effective therapy for the treatment of XLH is the use of the FGF23-blocking antibody, burosumab, which prevents FGF23 phosphaturic effects and improves serum Pi levels (8–10). Indeed, recent clinical trials conducted in children and adults with XLH show superior efficacy of burosumab in normalizing serum Pi levels and improving musculoskeletal symptoms compared with Pi supplements and vitamin D analog therapy (9–13). To date, FGF23-blocking therapy has been tested in only 2 adult patients with ARHR (14) and showed promising results.

Increased FGF23 production in hereditary hypophosphatemic disorders is caused by loss-of-function mutations of bone factors involved in bone mineralization, including Pi-regulating gene with homologies to endopeptidase (PHEX) in XLH (15) and dentin matrix protein 1 (DMP1) in ARHR type 1 (ARHR1) (5, 16). PHEX and DMP1 are both expressed in mature osteoblasts and osteocytes. PHEX is a membrane metalloendopeptidase that recognizes the conserved acidic serine–aspartate-rich matrix extracellular phosphoglycoprotein-associated motif of small integrin-binding ligand N-linked glycoproteins (SIBLING) in the extracellular space (17–19). DMP1 is an extracellular matrix protein that belongs to the SIBLING protein family and facilitates nucleation of hydroxyapatite during mineralization (20). Mutations in PHEX and DMP1 result in overlapping phenotypes in humans (5, 15, 16) and in mice (21, 22).

Multiple preclinical studies, using *Phex*-deficient Hyp mice (murine homolog of XLH) and *Dmp1*-null mice (murine homolog of ARHR1), have demonstrated the crucial role of FGF23 in hereditary hypophosphatemic disorders. Several studies showed that the global deletion of *Fgf23* in Hyp mice, resulting in hyperphosphatemia, hypervitaminosis D, and early mortality, reverted the phenotype of Hyp mice to a bone phenotype resembling that of *Fgf23*-null mice (23, 24). The global deletion of *Fgf23* in *Dmp1*-null mice showed similar limitations due to Pi and vitamin D toxicity (25). With developing genetic engineering tools, a more recent study overcame the limitations of the global deletion of *Fgf23* using instead a conditional deletion of *Fgf23* in osteoblasts and osteocytes of Hyp mice in order to normalize, but not fully suppress, FGF23 levels (26). This approach completely restored Pi levels, bone growth, and mineralization, further demonstrating the major role of FGF23 and Pi bioavailability in the pathogenesis of rickets and osteomalacia.

In the present study, we investigated the independent roles of Pi, FGF23, and DMP1 in the pathogenesis of ARHR, using dietary Pi supplementation and the osteocyte-specific deletion of *Fgf23* in *Dmp1*-null mice. We hypothesized that reduction of osteocyte-produced FGF23 in *Dmp1*-null mice might lead to correction of hypophosphatemia and improve bone growth and mineralization, similar to Hyp mice. We studied the bone and mineral phenotype of 12-week-old adult mice and further investigated the direct effects of FGF23 excess and DMP1 deficiency on osteoblast differentiation and activity using primary osteoblast cultures and transcriptomics.

## Results

### Dietary Pi supplementation aggravates FGF23 excess, rickets, and osteomalacia in adult Dmp1^KO^ mice.

**Figure 1:**
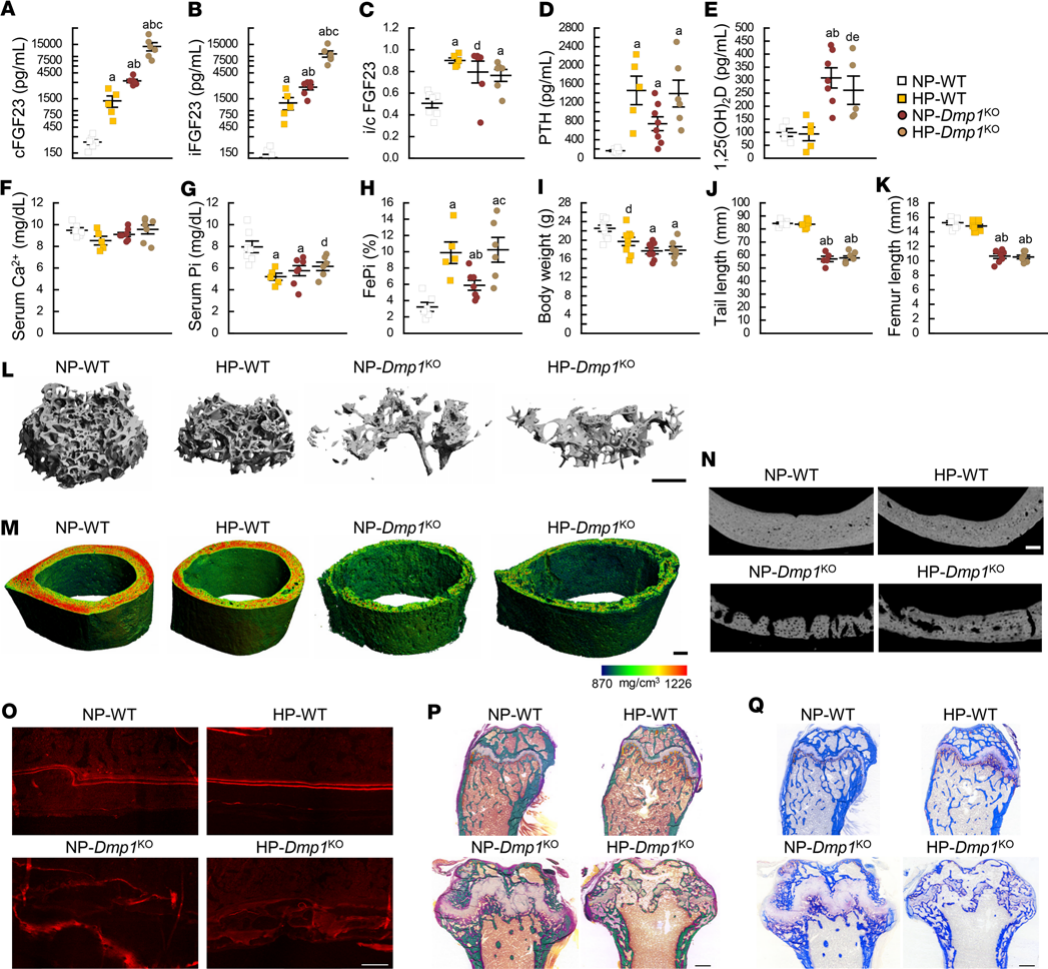
Dietary phosphate supplementation aggravates FGF23 excess and bone microarchitecture in *Dmp1*^KO^ mice. Serum levels of (**A**) total FGF23 (cFGF23), (**B**) intact FGF23 (iFGF23), (**C**) intact to total FGF23 ratio (i/c FGF23), (**D**) parathyroid hormone (PTH), (**E**) 1,25-dihydroxyvitamin D [1,25(OH)_2_D], (**F**) calcium (Ca^2+^), and (**G**) phosphate (Pi); (**H**) fractional excretion of Pi (FePi); (**I**) body weight, (**J**) tail length, and (**K**) femur length; 3D-μCT scan reconstruction of (**L**) distal femur trabecular metaphysis (scale bar = 200 μm); (**M**) midshaft femur cortical diaphysis (scale bar = 500 μm); (**N**) 2D μCT analysis of cortical bone porosity (scale bar = 100 μm); (**O**) red fluorescence microscopy imaging of alizarin red S–stained (ARS-stained) mineralization fronts; (**P**) bright-field microscopy imaging of modified trichrome Goldner staining; and (**Q**) tartrate-resistant acidic phosphatase (TRAcP) staining of longitudinal histology sections of distal femur (scale bar = 100 μm for ARS, 500 μm for Goldner and TRAcP). All analyses were performed in 12-week-old WT (*n* ≥ 5) and *Dmp1*^KO^ (*n* ≥ 5) mice fed a diet containing 0.7% Pi (normal Pi, NP) or 2% Pi (high Pi, HP) from 6 to 12 weeks of age. Values are expressed as mean ± SEM; *P* < 0.05 vs. ^a^NP-WT, ^b^HP-WT, ^c^NP-*Dmp1*^KO^; *P* < 0.1 vs. ^d^NP-WT, ^e^HP-WT. Statistical tests were ANOVA test followed by post hoc *t* tests and multiple-testing correction using Holm-Bonferroni method.

Oral Pi additives are one of the main components of the conventional therapy used in the treatment of hereditary hypophosphatemia. We first assessed the effects of dietary Pi supplementation in WT and *Dmp1*^KO^ mice by feeding them a diet that contains either 0.7% Pi (normal Pi, NP) or 2% Pi (high Pi, HP) from 6 to 12 weeks of age. At 12 weeks, HP-WT mice showed increased serum total FGF23 (cFGF23), intact FGF23 (iFGF23), and parathyroid hormone (PTH) levels that resulted in increased fractional excretion of Pi (FePi) compared with NP-WT mice (Figure 1, A–H). The i/c FGF23 ratio also increased in HP-WT mice, suggesting a reduction in FGF23 cleavage. Because mice were fasted overnight prior to blood collection, we did not observe hyperphosphatemia in HP-WT, and instead, HP-WT showed low serum Pi levels likely driven by the overnight phosphaturic action of increased iFGF23 in the absence of sustained Pi supply. Six weeks of HP diet did not significantly influence skeletal growth (Figure 1, I–K, and Supplemental Figure 1A; supplemental material available online with this article; https://doi.org/10.1172/jci.insight.156850DS1). However, HP-WT mice showed reduced trabecular and cortical bone mass (Figure 1, L–N, and Supplemental Figure 1, B–O) likely due to increased PTH levels. In accordance with previous studies (5, 22), DMP1 deficiency in *Dmp1*^KO^ mice increased serum cFGF23, iFGF23, and i/c FGF23 levels, which led to increased FePi, resulting in hypophosphatemia. At 12 weeks, *Dmp1*^KO^ mice also displayed hyperparathyroidism and a surprising 3-fold increase in 1,25(OH)_2_D levels (Figure 1, A–H). In *Dmp1*^KO^ mice, 6 weeks of dietary Pi supplementation exacerbated the preexisting cFGF23 and iFGF23 excess, leading to a further increase in FePi. Body weight, tail length, and femur length were reduced at the same level in NP- and HP-*Dmp1*^KO^ mice (Figure 1, I–K). Consistent with previous studies performed in younger *Dmp1*^KO^ mice, bone growth was partially improved only when dietary Pi supplementation started in utero or at birth (Supplemental Figure 1A). When started at 6 weeks of age, HP diet induced further reductions in trabecular thickness and bone mineral density and radial expansion of the diaphysis compared with NP-*Dmp1*^KO^ (Supplemental Figure 1, B–O). 2D bone histology analyses showed similar effects concomitant with a severe reduction of the growth plate in HP-*Dmp1*^KO^ compared with NP-*Dmp1*^KO^ mice (Figure 1, O–Q). Together, this supports an overall worsening of the ricketic phenotype and osteomalacia in adult *Dmp1*^KO^ mice fed an HP diet for 6 weeks.

### Reduction of FGF23 levels fully corrects hypophosphatemia in Dmp1^KO^ mice.

**Figure 2:**
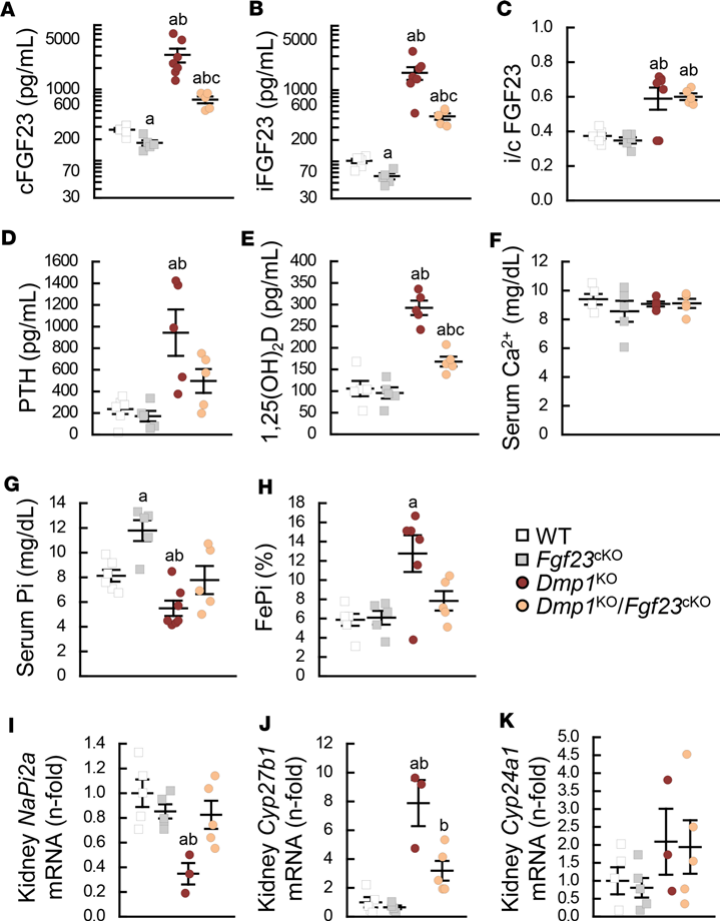
Osteocyte-specific deletion of *Fgf23* fully corrects hypophosphatemia in *Dmp1*^KO^ mice. Serum levels of (**A**) total FGF23 (cFGF23), (**B**) intact FGF23 (iFGF23), (**C**) intact to total FGF23 ratio (i/c FGF23), (**D**) parathyroid hormone (PTH), (**E**) 1,25-dihydroxyvitamin D [1,25(OH)_2_D], (**F**) calcium (Ca^2+^), and (**G**) phosphate (Pi); (**H**) fractional excretion of Pi (FePi); and kidney mRNA expression of (**I**) *NaPi2a*, (**J**) *Cyp27b1*, and (**K**) *Cyp24a1* in 12-week-old WT (*n* ≥ 5), *Fgf23*^Dmp1-cKO^ (*n* ≥ 5), *Dmp1*^KO^ (*n* ≥ 3), and *Dmp1*^KO^ *Fgf23*^Dmp1-cKO^ (*n* ≥ 5) mice. Values are expressed as mean ± SEM; *P* < 0.05 vs. ^a^WT, ^b^*Fgf23*^cKO^, ^c^*Dmp1*^KO^. Statistical tests were ANOVA test followed by post hoc *t* tests and multiple-testing correction using Holm-Bonferroni method.

In hereditary rickets, FGF23 is produced in excess by bone, resulting in increased serum FGF23 levels that increase renal Pi excretion and cause hypophosphatemia. To understand the contribution of bone-produced FGF23 in the pathology of DMP1 deficiency, we next deleted *Fgf23* in mature osteoblasts and osteocytes, using a *Dmp1*-cre recombinase, in WT and *Dmp1*^KO^ mice. Consistent with successful *Fgf23* deletion (Supplemental Figure 2A), *Dmp1^+/+^* *Fgf23^fl/fl^* *Dmp1-cre^+^* (*Fgf23*^cKO^) mice showed a 40% reduction in bone *Fgf23* mRNA and serum cFGF23 and iFGF23 levels while i/c FGF23 remained unchanged compared to WT mice (Figure 2, A–C, and Supplemental Figure 2B). In response to lower FGF23 levels, serum Pi levels were increased in *Fgf23*^cKO^ mice, and serum PTH and 1,25(OH)_2_D levels remained unchanged compared to WT mice (Figure 2, D–H). As expected, cFGF23, iFGF23, i/c FGF23, and PTH levels were increased in *Dmp1*^KO^ mice. This was associated with reduced kidney sodium-dependent phosphate transport protein 2A (*NaPi2a*) expression and increased FePi, resulting in hypophosphatemia (Figure 2, A–I). Once again 12-week-old *Dmp1*^KO^ mice displayed a 3-fold increase in 1,25(OH)_2_D levels that was associated with increased kidney *Cyp27b1* expression (Figure 2, J and K). Osteocyte-specific deletion of *Fgf23* in *Dmp1*^KO^ mice corrected bone *Fgf23* mRNA (Supplemental Figure 2B) and reduced cFGF23 and iFGF23 excess by approximately 75%. In contrast, i/c FGF23 remained elevated in *Dmp1*^KO^ *Fgf23*^cKO^ mice, consistent with reduced FGF23 cleavage in the absence of DMP1 (21). *Dmp1*^KO^ *Fgf23*^cKO^ mice also showed a 50% reduction in serum PTH and a proportional reduction in 1,25(OH)_2_D levels compared with *Dmp1*^KO^ mice. The combined reduction in iFGF23 and PTH normalized serum Pi levels and FePi. Of note, calcium levels remained unchanged in all groups (Figure 2, A–K).

### Reduction of FGF23 levels partially corrects bone growth and mineralization in Dmp1^KO^ mice.

**Figure 3:**
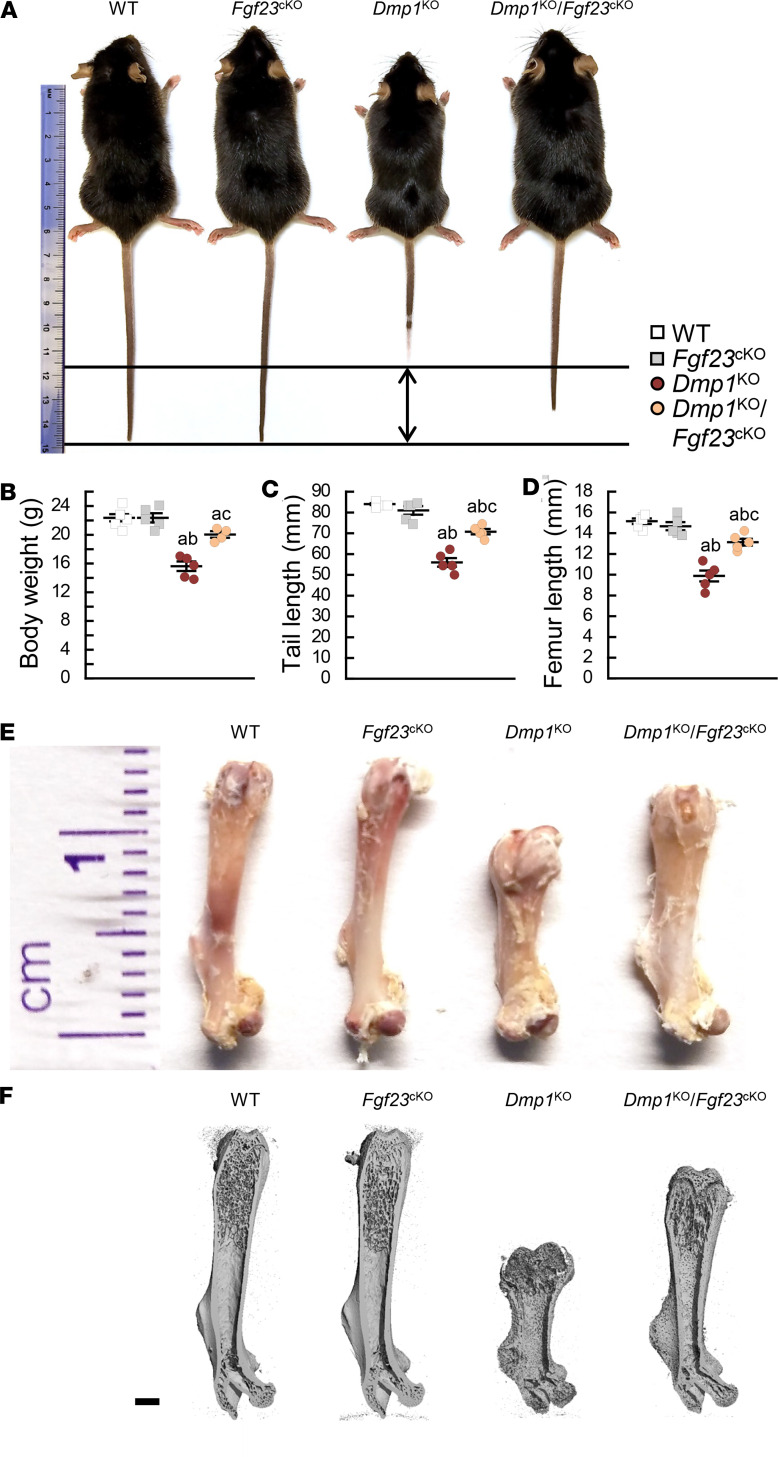
Osteocyte-specific deletion of *Fgf23* partially corrects bone growth in *Dmp1*^KO^ mice. (**A**) Mouse gross appearance, (**B**) body weight, (**C**) tail length, (**D**) femur length, (**E**) femur gross appearance, and (**F**) 3D μCT representation of total femur in sagittal plane (scale bar = 1 mm) in 12-week-old WT (*n* ≥ 6), *Fgf23*^Dmp1-cKO^ (*n* ≥ 5), *Dmp1*^KO^ (*n* ≥ 5), and *Dmp1*^KO^ *Fgf23*^Dmp1-cKO^ (*n* ≥ 4) mice. Values are expressed as mean ± SEM; *P* < 0.05 vs. ^a^WT, ^b^*Fgf23*^cKO^, ^c^*Dmp1*^KO^. Statistical tests were performed using ANOVA test followed by post hoc *t* tests and multiple-testing correction using Holm-Bonferroni method.

**Figure 4:**
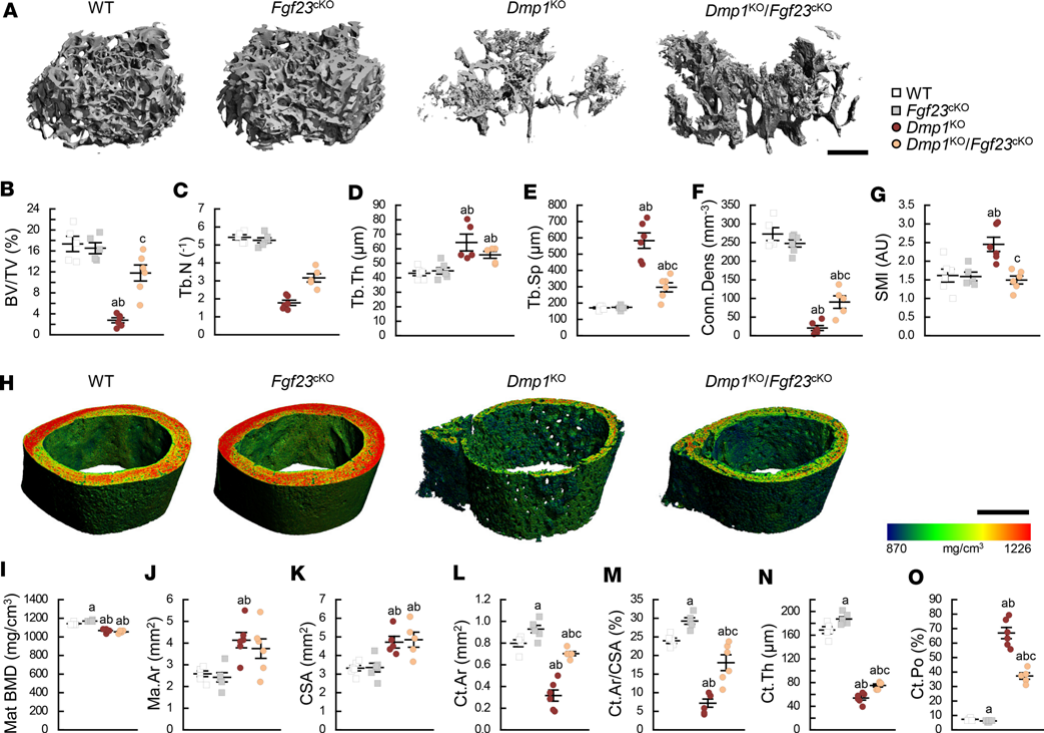
Osteocyte-specific deletion of *Fgf23* partially restores bone microarchitecture in *Dmp1*^KO^ mice. 3D μCT (**A**) scan reconstruction of distal femur trabecular metaphysis (scale bar = 500 μm) and (**B**–**G**) parameters of trabecular bone microarchitecture; (**H**) scan reconstruction of midshaft femur cortical diaphysis (scale bar = 500 μm) and (**I**–**O**) parameters of cortical bone microarchitecture. All analyses were performed in 12-week-old WT (*n* ≥ 5), *Fgf23*^Dmp1-cKO^ (*n* ≥ 5), *Dmp1*^KO^ (*n* ≥ 5), and *Dmp1*^KO^ *Fgf23*^Dmp1-cKO^ (*n* ≥ 5) mice. Values are expressed as mean ± SEM; *P* < 0.05 vs. ^a^WT, ^b^*Fgf23*^cKO^, ^c^*Dmp1*^KO^. Statistical tests were ANOVA test followed by post hoc *t* tests and multiple-testing correction using Holm-Bonferroni method. BV/TV, bone volume to tissue volume ratio; Tb.N, trabecular number; Tb.Th, trabecular thickness; Tb.Sp, trabecular separation; Conn.Dens, connectivity density; SMI, structural model index; mat BMD, material bone mineral density; Ma.Ar, marrow area; CSA, cross-sectional area; Ct.Ar, cortical area; Ct.Th, cortical thickness; Ct.Po, cortical porosity.

**Figure 5:**
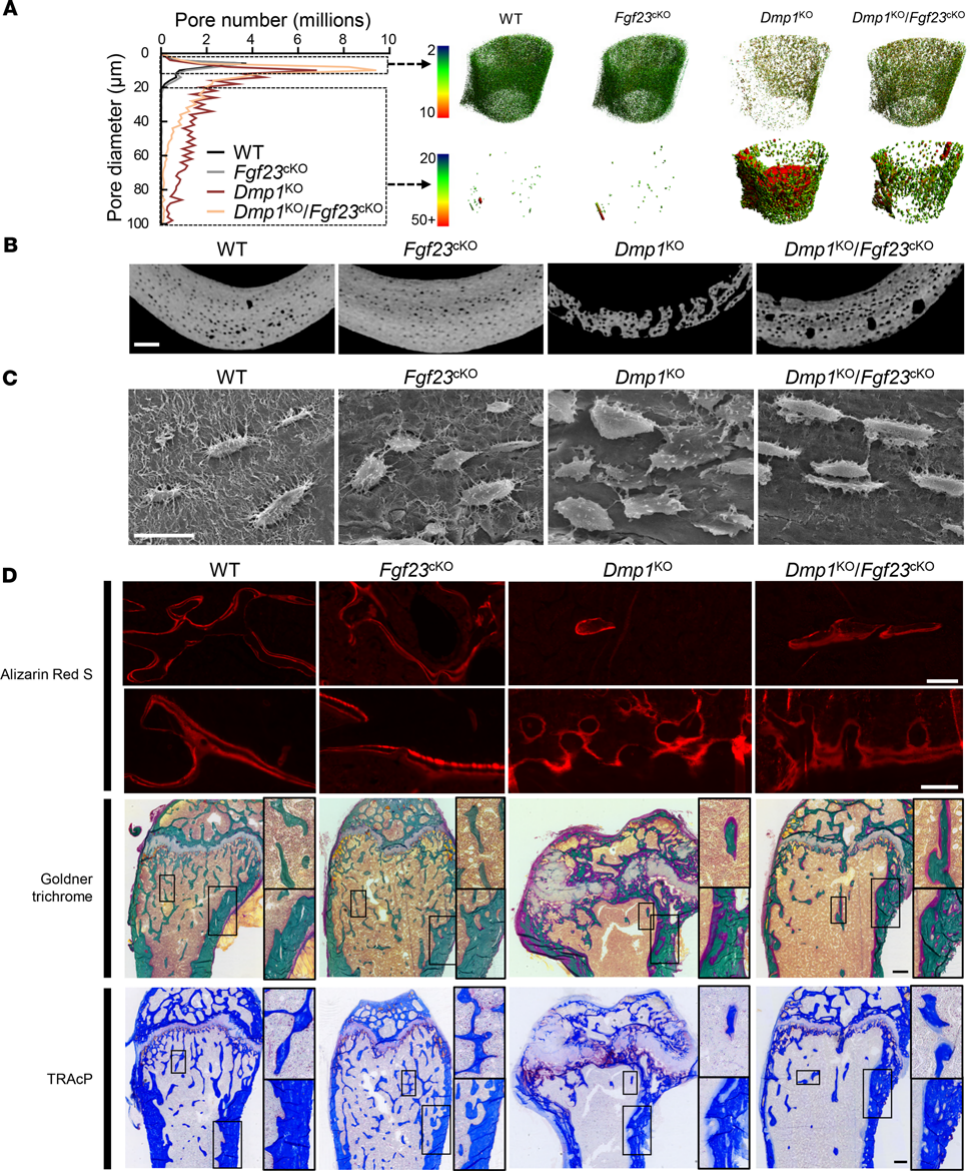
Osteocyte-specific deletion of *Fgf23* partially restores mineralization and lacuno-canalicular network in *Dmp1*^KO^ mice. (**A** and **B**) High-resolution μCT analysis of cortical bone porosity (scale bar = 100 μm), (**C**) acid-etched scanning electron microscopy of femur cortical bone (scale bar = 20 μm), (**D**) red fluorescence microscopy imaging of ARS-stained mineralization fronts (top), and bright-field microscopy imaging of modified trichrome Goldner staining (middle) and tartrate-resistant acidic phosphatase (TRAcP) staining (bottom) of longitudinal histology sections of distal femur (scale bar = 100 μm for ARS, 250 μm for Goldner and TRAcP). For each staining, top (×3.5 original magnification) and bottom (×1.8 original magnification) zoom-in panels represent regions of interest in trabecular and in cortical bone, respectively. All analyses were performed in 12-week-old WT (*n* ≥ 5), *Fgf23*^Dmp1-cKO^ (*n* ≥ 5), *Dmp1*^KO^ (*n* ≥ 5), and *Dmp1*^KO^ *Fgf23*^Dmp1-cKO^ (*n* ≥ 5) mice.

WT and *Fgf23*^cKO^ mice were similar in growth and appearance (Figure 3) and displayed similar trabecular bone microarchitecture (Figure 4, A–G); however, *Fgf23*^cKO^ mice showed increased cortical bone mass compared with WT mice (Figure 4, H–O, and Supplemental Table 1). *Dmp1*^KO^ mice were smaller than WT mice and showed a 30%–35% reduction in body weight and tail and femur lengths, along with larger epiphyses, typical features of growth retardation and rickets (Figure 3). As shown in prior studies (21, 22), *Dmp1*^KO^ mice displayed reduced mineralized trabecular bone volume due to osteomalacia (Figure 4, A–G) and rickets-associated cortical bone expansion and impaired mineralization (Figure 4, H–O, and Supplemental Table 1). In contrast with 6 weeks of dietary Pi supplementation, bone-specific deletion of *Fgf23* in *Dmp1*^KO^ mice partially corrected body weight and tail and femur lengths (Figure 3 and Supplemental Figure 2C). Despite these corrections, *Dmp1*^KO^ *Fgf23*^cKO^ mice also showed residual features of rickets, including larger epiphyses (Figure 3, E and F). Osteocyte-specific deletion of *Fgf23* in *Dmp1*^KO^ mice also resulted in a partial rescue of most trabecular and cortical bone defects, with the exception of cortical bone expansion (Figure 4 and Supplemental Table 1), consistent with residual features of rickets (Figure 3). In *Dmp1*^KO^ mice, osteomalacia is associated with altered osteocyte morphology and enlarged osteocyte lacunae (27), also shown in 3D μCT analyses by increased cortical bone porosity. High-resolution μCT scanning and scanning electron microscopy (SEM) analyses of cortical bone showed that osteocyte-specific deletion of *Fgf23* in *Dmp1*^KO^ mice resulted in smaller pore diameter and partially improved cortical bone porosity compared with *Dmp1*^KO^ mice. Despite ameliorations of the cortical bone phenotype, some alterations in osteocyte morphology persisted in compound *Dmp1*^KO^ *Fgf23*^cKO^ mice, including the apparent loss of connectivity via canaliculi (Figure 5, A–C). We also observed a partial correction of the bone phenotype by 2D histology analyses. *Dmp1*^KO^ *Fgf23*^cKO^ mice showed improved growth plate anatomy and better defined ARS seams compared with *Dmp1*^KO^ mice. However, they also showed residual osteoid buildup, indicating impaired mineralization (Figure 5D). Osteoclast number was significantly reduced in all mutant groups compared with WT mice (Supplemental Table 4), suggesting that neither reduction of FGF23 nor Pi increase corrected aberrant bone resorption. Of note, the overall phenotype of WT *Fgf23^+/+^* *Dmp1-cre^+^* and *Dmp1*^KO^ *Fgf23^+/+^* *Dmp1-cre^+^* mice is similar to WT *Fgf23^fl/fl^* *Dmp1-cre^–^* and *Dmp1*^KO^ *Fgf23^fl/fl^* *Dmp1-cre^–^* used in our study (Supplemental Figure 3 and Supplemental Tables 2 and 3), suggesting that the expression of the *Dmp1*-cre in the absence of the floxed *Fgf23* allele is not responsible for phenotypic changes.

### Differential effects of Pi, FGF23, and DMP1 on osteoblast differentiation and mineralization.

**Figure 6:**
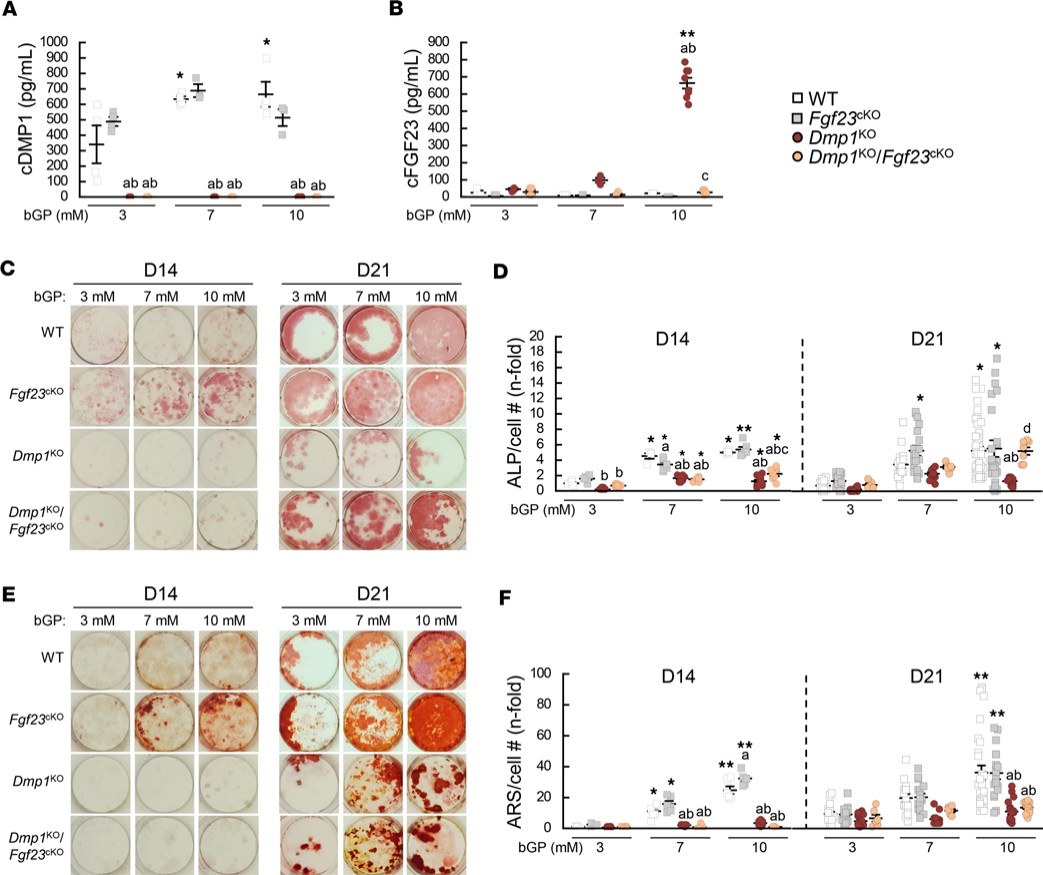
Osteocyte-specific deletion of *Fgf23* restores osteoblast differentiation but does not restore impaired mineralization in *Dmp1*^KO^ primary osteoblast cultures. Bone marrow stromal cells were isolated from 12-week-old WT (*n* ≥ 3), *Fgf23*^Dmp1-cKO^ (*n* ≥ 3), *Dmp1*^KO^ (*n* ≥ 3), and *Dmp1*^KO^ *Fgf23*^Dmp1-cKO^ (*n* ≥ 4) mice, then cultured for 14 (D14) and 21 (D21) days in osteoblast differentiation medium containing 3, 7, or 10 mM of beta-glycerophosphate (bGP). Levels of (**A**) total DMP1 (cDMP1) and (**B**) total FGF23 (cFGF23) in conditioned media collected at D21. (**C** and **D**) Alkaline phosphatase (ALP) staining and quantification and (**E** and **F**) alizarin red S (ARS) staining and quantification. Values are expressed as mean ± SEM; *P* < 0.05 vs. bGP treatment–matched ^a^WT, ^b^*Fgf23*^cKO^, ^c^*Dmp1*^KO^, and genotype-matched *3 mM and **7 mM within each time point. ^d^*P* = 0.1 vs. 10 mM *Dmp1*^KO^. Statistical tests were ANOVA test followed by Bonferroni post hoc tests.

To understand the direct effects of Pi, FGF23, and DMP1 on osteoblasts, in the absence of systemic changes, we cultured primary osteoblasts isolated from WT, *Fgf23*^cKO^, *Dmp1*^KO^, and *Dmp1*^KO^ *Fgf23*^cKO^ mice. In all cultures, we used osteoblast differentiation medium containing incremental concentrations of beta-glycerophosphate (bGP) and assessed the secretion of FGF23 and DMP1 in the medium, as well as osteoblast differentiation and activity after 14 and 21 days of culture. After 21 days, DMP1 protein levels were similar in WT and *Fgf23*^cKO^ culture media and undetectable in *Dmp1*^KO^ and *Dmp1*^KO^ *Fgf23*^cKO^ cultures (Figure 6A). FGF23 levels increased only in the *Dmp1*^KO^ cultures in the presence of 10 mM bGP, and this was fully corrected by the osteocyte-specific deletion of *Fgf23* (Figure 6B). WT and *Fgf23*^cKO^ osteoblasts showed a dose- and time-dependent increase in differentiation, assessed by alkaline phosphatase (ALP) staining, that peaked at day 21. Interestingly, peak differentiation was achieved using 10 mM bGP in WT cells and only 7 mM bGP in *Fgf23*^cKO^ cells (Figure 6, C and D). *Dmp1*^KO^ cultures showed reduced ALP staining at all bGP concentrations and time points. This was fully rescued at day 21 in 10 mM bGP *Dmp1*^KO^ *Fgf23*^cKO^ cultures, suggesting that FGF23 excess inhibits the differentiation of *Dmp1*^KO^ osteoblasts. WT and *Fgf23*^cKO^ osteoblasts also showed a dose- and time-dependent increase in osteoblast activity and mineralization, assessed by ARS staining, that peaked with 10 mM bGP at day 21 in both groups (Figure 6, E and F). *Dmp1*^KO^ cultures showed reduced ARS staining at all bGP concentrations and time points. While deletion of *Fgf23* partially restored osteoblastic differentiation in *Dmp1*^KO^ *Fgf23*^cKO^ cultures, ARS staining was similar in *Dmp1*^KO^ and *Dmp1*^KO^ *Fgf23*^cKO^ cultures. This suggests that despite adequate Pi availability, DMP1 deficiency, but not FGF23 excess, contributes to abnormal mineral apposition.

### FGF23 excess contributes to reduced differentiation of Dmp1^KO^ osteoprogenitors.

**Figure 7:**
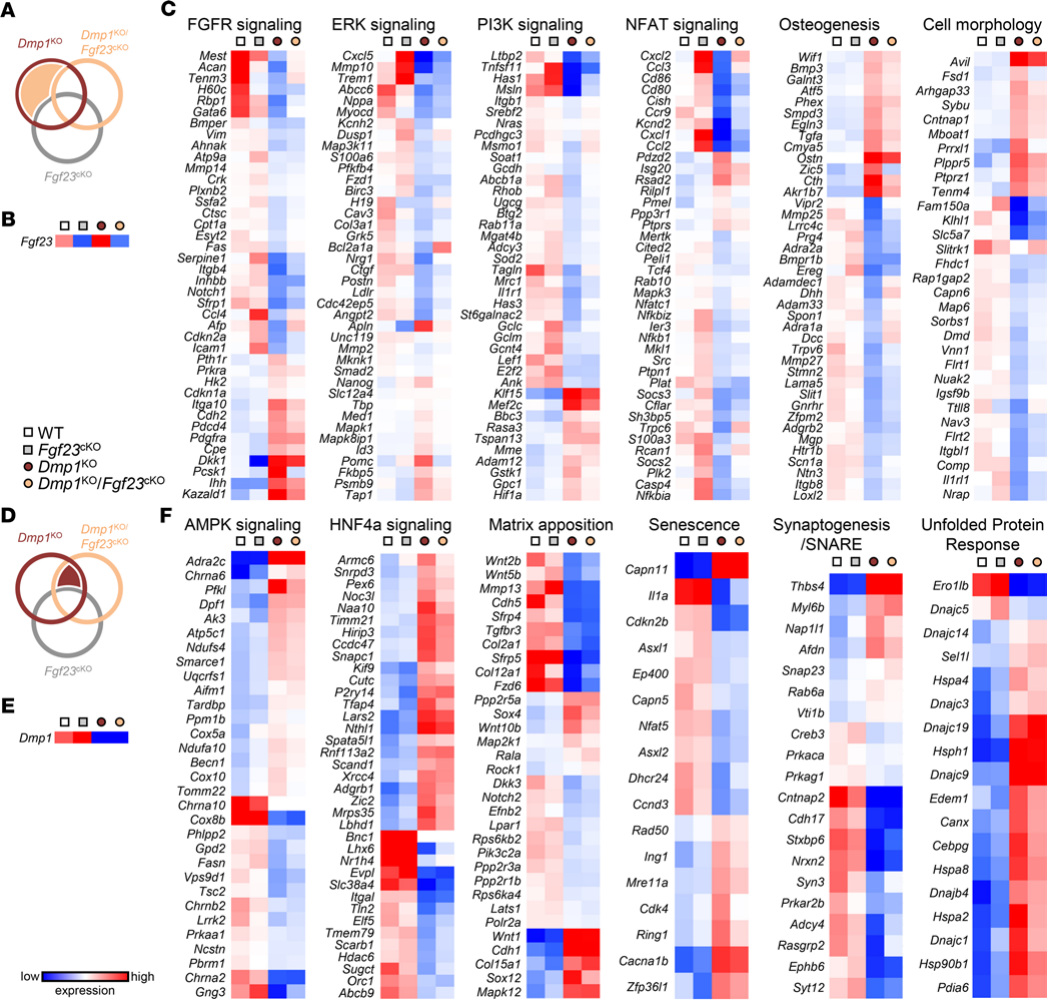
Canonical pathways altered in *Dmp1*^KO^ osteoblasts are differentially regulated by FGF23 and by DMP1. Bulk RNA-sequencing analysis was performed on bone marrow stromal cells isolated from 12-week-old WT (*n* = 3), *Fgf23*^Dmp1-cKO^ (*n* = 3), *Dmp1*^KO^ (*n* = 3), and *Dmp1*^KO^ *Fgf23*^Dmp1-cKO^ (*n* = 3) mice and cultured for 21 days in osteoblast differentiation medium containing 10 mM of beta-glycerophosphate. (**A**) Venn diagram identifies genes showing altered expression in *Dmp1*^KO^ but not in *Dmp1*^KO^ *Fgf23*^cKO^ osteoblasts (*tan area*). Heatmaps represent the expression of (**B**) *Fgf23* and (**C**) genes identified in **A** and used in Ingenuity Pathway Analysis (IPA; QIAGEN) to define the most represented canonical pathways regulated by FGF23. (**D**) Venn diagram identifies genes showing altered expression in *Dmp1*^KO^ and in *Dmp1*^KO^ *Fgf23*^cKO^ osteoblasts (*burgundy area*). Heatmaps represent the expression of (**E**) *Dmp1* and (**F**) genes identified in **D** and used in IPA to define the most represented canonical pathways regulated by DMP1. Statistical tests were unpaired *t* test and corrected by the false discovery rate (*P* < 0.1).

To investigate the mechanisms involved in altered osteoblast differentiation and mineralization, we performed bulk RNA sequencing (RNA-Seq) and subsequent canonical pathway analyses in 10 mM bGP cultures at day 21. To identify the pathways specifically regulated by Fgf23 in *Dmp1*^KO^ osteoblasts, we performed a pathway analysis on 4,094 genes showing altered expression in *Dmp1*^KO^ but not in *Fgf23*^cKO^ or *Dmp1*^KO^ *Fgf23*^cKO^ osteoblasts (Figure 7A). We verified reduced *Fgf23* mRNA in both *Fgf23*^cKO^ and *Dmp1*^KO^ *Fgf23*^cKO^ osteoblasts, and increased *Fgf23* mRNA in *Dmp1*^KO^ osteoblasts, compared with WT cells (Figure 7B). Altered FGF receptor (FGFR), ERK, PI3K, and nuclear factor of activated T cells (NFAT) signaling, and osteogenesis and cell morphology were the most regulated pathways represented in this set (Figure 7C). These pathways were no longer identified in a second analysis performed on 1,547 genes showing altered expression in both *Dmp1*^KO^ and *Dmp1*^KO^ *Fgf23*^cKO^ osteoblasts (Figure 7D). Among these genes, we verified reduced *Dmp1* mRNA in *Dmp1*^KO^ and *Dmp1*^KO^ *Fgf23*^cKO^ compared with WT and *Fgf23*^cKO^ osteoblasts (Figure 7E). Altered AMPK and hepatocyte nuclear factor 4α (HNF4α) signaling were the most regulated intracellular signaling pathways represented in this analysis, along with altered matrix apposition, senescence, synaptogenesis, and unfolded protein response (Figure 7F). Together, this supports differential effects of FGF23 excess and DMP1 deficiency on osteoblast differentiation and mineralization, respectively.

**Figure 8:**
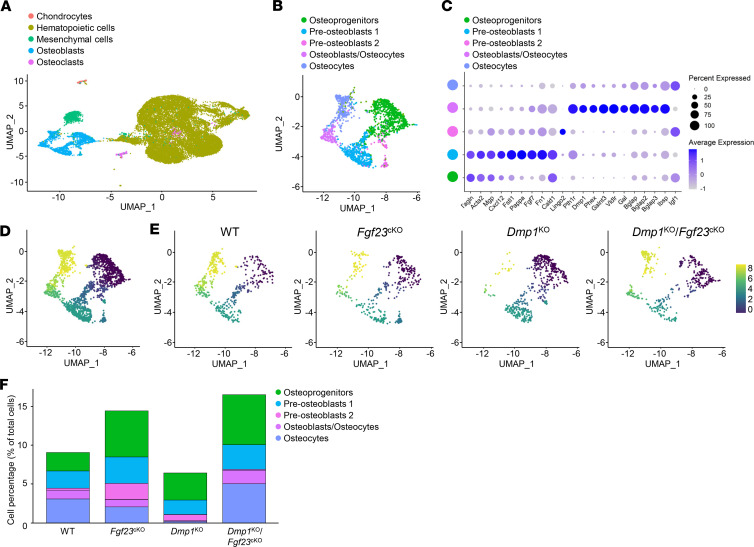
Osteocyte-specific deletion of *Fgf23* restores osteoblast differentiation in *Dmp1*^KO^ at the single-cell level. Single-cell RNA-sequencing analysis was performed on bone marrow stromal cells isolated from 12-week-old WT (*n* = 3), *Fgf23*^Dmp1-cKO^ (*n* = 3), *Dmp1*^KO^ (*n* = 3), and *Dmp1*^KO^ *Fgf23*^Dmp1-cKO^ (*n* = 3) mice and cultured for 21 days in osteoblast differentiation medium containing 10 mM of beta-glycerophosphate. Uniform manifold approximation and projection (UMAP) plot of (**A**) total isolated cells and (**B**) osteoblast lineage cells only. (**C**) Dot plot of cluster-enriched markers in osteoblastic cells segregated by clusters of differentiation. Pseudotime analysis of osteoblastic cluster from (**D**) all groups combined and (**E**) segregated by group. (**F**) Percentage of osteoblast-like cells in the total number of isolated cells segregated by cluster of differentiation and by group.

**Figure 9:**
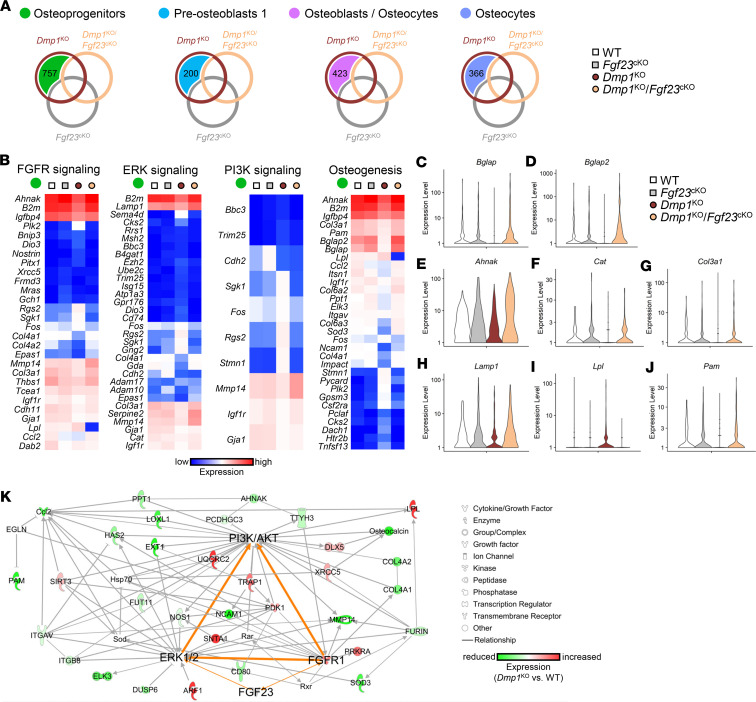
FGF23 targets osteoprogenitors via FGFR/ERK/PI3K signaling. Single-cell RNA-sequencing analysis was performed on bone marrow stromal cells isolated from 12-week-old WT (*n* = 3), *Fgf23*^Dmp1-cKO^ (*n* = 3), *Dmp1*^KO^ (*n* = 3), and *Dmp1*^KO^ *Fgf23*^Dmp1-cKO^ (*n* = 3) mice and cultured for 21 days in osteoblast differentiation medium containing 10 mM of beta-glycerophosphate. (**A**) Venn diagram identifies genes showing altered expression in *Dmp1*^KO^ but not in *Dmp1*^KO^ *Fgf23*^cKO^ osteoblasts (*colored area*) in each cluster of differentiation. (**B**) Heatmaps represent the expression of genes identified in **A** in the osteoprogenitor cluster (*green dot*) and used in Ingenuity Pathway Analysis (IPA) to define the most represented canonical pathways regulated by FGF23. (**C**–**J**) Violin plots representing the expression of most regulated target genes in *Dmp1*^KO^ and corrected in *Dmp1*^KO^ *Fgf23*^cKO^ osteoprogenitors. (**K**) IPA gene network analysis showing most connected gene targets in the osteoprogenitor cluster and identifying FGF receptor 1 (FGFR1), ERK1/2, and PI3K/AKT as common regulators of these targets. Statistical tests were Mann-Whitney’s *U* test and corrected by the false discovery rate (*P* < 0.1).

We next performed single-cell RNA-Seq (scRNA-Seq) analyses in 10 mM bGP WT, *Fgf23*^cKO^, *Dmp1*^KO^, and *Dmp1*^KO^ *Fgf23*^cKO^ cultures at day 21 to identify the most likely cell targets of FGF23 and DMP1. In total, we obtained 22 clusters (Supplemental Figure 4A) that we regrouped based on cell identity into 5 parent populations, including chondrocyte-, hematopoietic-, mesenchymal-, osteoblast- and osteoclast-like cells (Figure 8A). Based on the expression of specific osteoblast markers (Supplemental Figure 4, B–M), we identified 5 clusters of osteoblastic cells, including osteoprogenitors, pre-osteoblasts 1 and 2, osteoblasts/osteocytes, and osteocytes (Figure 8, B and C). These were also delineated by pseudotime analysis showing the progressive differentiation of osteoblasts from osteoprogenitors to osteocytes (Figure 8D). When segregated by group, the pseudotime analysis shows that in *Dmp1*^KO^ cultures, the number of osteoprogenitors and pre-osteoblasts was increased, the pre-osteoblast 2 cluster was quasi-absent, and the number of mature osteoblasts and osteocytes was reduced compared with all other groups (Figure 8, E and F). Of note, *Fgf23* was not detected probably because of current known limitations of droplet-based scRNA-Seq expression profiling pipelines using short coverage of mRNA from the 3′UTR end, which fails to detect low, yet specific, gene expression in less prevalent cell populations (28–30). Interestingly, *Fgf23*^cKO^ and *Dmp1*^KO^ *Fgf23*^cKO^ cultures showed the highest, and *Dmp1*^KO^ the lowest, number of cells in the osteoblastic parent cluster proportionally to the total number of cultured cells (Figure 8F). Similar to bulk RNA-Seq analyses, in each subcluster, we selected the genes that showed altered expression in *Dmp1*^KO^ but not in *Fgf23*^cKO^ or *Dmp1*^KO^ *Fgf23*^cKO^ cells (Figure 9A) and performed a canonical pathway analysis on each data set. The osteoprogenitor cluster showed the highest number of altered genes (757 genes), and altered FGFR, ERK, and PI3K signaling and osteogenesis were the most represented pathways (Figure 9B). Among the 757 selected genes, *Bglap*, *Ahnak*, *Cat*, *Col3a1*, *Lamp1*, *Lpl*, and *Pam* were representative of genes showing altered expression in *Dmp1*^KO^ that was corrected in *Dmp1*^KO^ *Fgf23*^cKO^ osteoprogenitors (Figure 9, C–J). Gene network analysis of the 757 genes identified additional gene targets that shared the most regulatory interactions, and these connected via 3 main hub-like nodes, including FGFR1, ERK1/2, and PI3K/AKT signaling, regulated upstream by FGF23 (Figure 9K). These target genes and pathways were not identified as the most regulated targets in the other clusters (data not shown), demonstrating that FGF23 specifically targets osteoprogenitors in *Dmp1*^KO^ cells. We performed a similar analysis on genes showing common alterations in *Dmp1*^KO^ and *Dmp1*^KO^ *Fgf23*^cKO^ osteoblasts to determine DMP1-specific targets (Supplemental Figure 5). The osteocyte subcluster showed the highest number of genes (506 genes) altered similarly in both groups, leading to changes in AMPK and HNF4α signaling and matrix apposition and senescence.

### FGF23 excess directly inhibits osteoblast differentiation.

**Figure 10:**
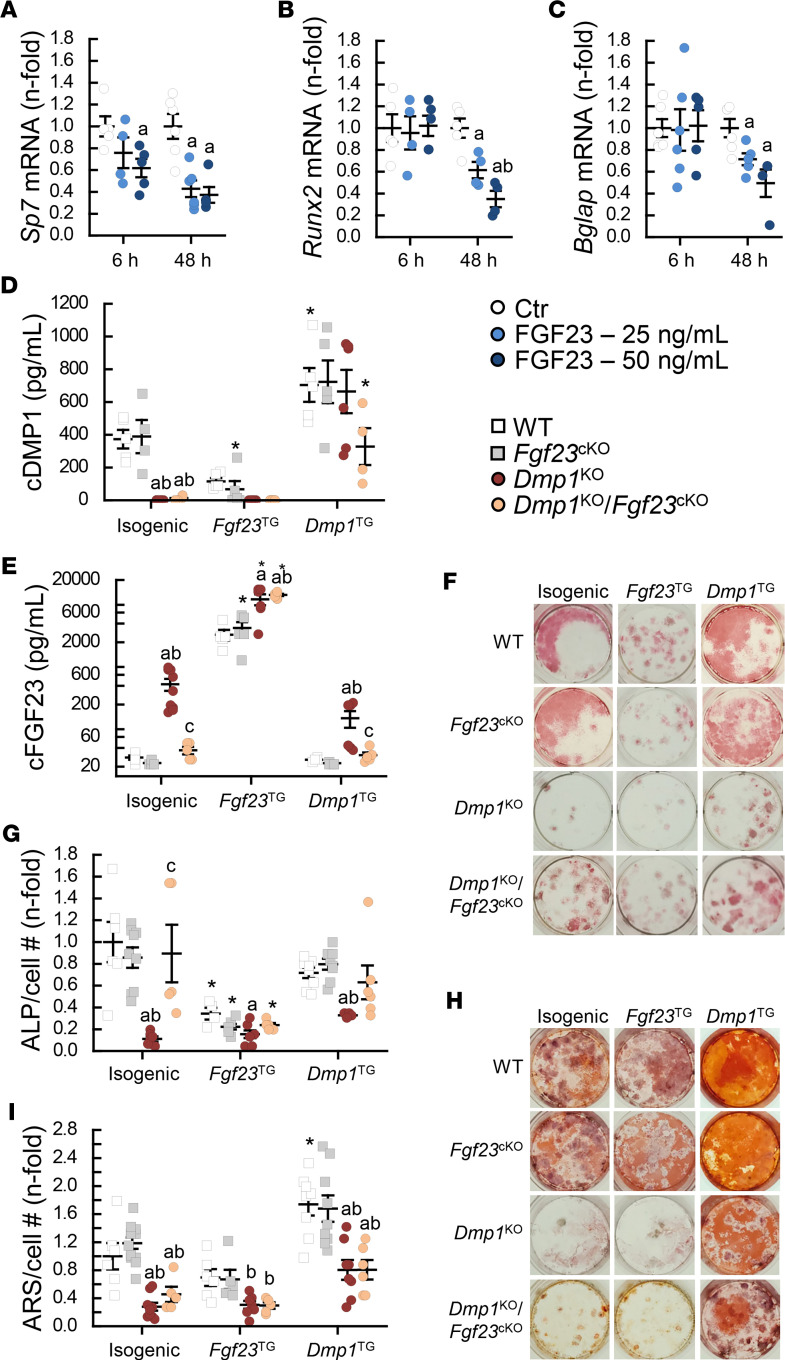
FGF23 directly inhibits osteoblast differentiation. (**A**–**C**) mRNA expression of markers of osteoblast differentiation in MC3T3-E1 osteoblasts cultured for 21 days and treated with recombinant FGF23 (0, 25, and 50 ng/mL) for the last 6 and 48 hours of culture (*n* ≥ 4). Levels of (**D**) total DMP1 (cDMP1) and (**E**) total FGF23 (cFGF23) measured by ELISA in conditioned culture media from bone marrow stromal cells (BMSCs) isolated from 12-week-old WT (*n* ≥ 4), *Fgf23*^Dmp1-cKO^ (*n* ≥ 5), *Dmp1*^KO^ (*n* ≥ 5), and *Dmp1*^KO^ *Fgf23*^Dmp1-cKO^ (*n* ≥ 5) mice, then cocultured for 21 days with BMSCs isolated from the same group (isogenic), or immortalized BMSCs displaying genetic overexpression of *Fgf23* (*Fgf23*^TG^) or *Dmp1* (*Dmp1*^TG^). (**F** and **G**) Alkaline phosphatase (ALP) staining and quantification and (**H** and **I**) alizarin red S (ARS) staining and quantification. Values are expressed as mean ± SEM; *P* < 0.05 vs. (**A**–**C**) time point–matched ^a^Ctr, ^b^FGF23 25 ng/mL, (**D**–**I**) culture-matched ^a^WT, ^b^*Fgf23*^cKO^, ^c^*Dmp1*^KO^, and *genotype-matched isogenic. Statistical tests were ANOVA test followed by post hoc *t* tests and multiple-testing correction using Holm-Bonferroni method (**A**–**C**) and by Bonferroni’s post hoc tests (**D**–**I**).

To show the direct effect of FGF23 on osteoblast differentiation, we cultured MC3T3-E1 osteoblasts for 21 days and treated them with escalating doses of FGF23 for the last 6 and 48 hours of culture. We showed that FGF23 significantly inhibited the expression of markers of osteoblast differentiation as early as 6 hours (*Sp7*) and after 48 hours of treatment (*Sp7*, *Runx2*, and *Bglap*) compared with control cells (Figure 10, A–C). We also tested the long-term effects of FGF23 and DMP1 on osteoblast differentiation and mineralization in primary osteoblasts isolated from WT, *Fgf23*^cKO^, *Dmp1*^KO^, and *Dmp1*^KO^ *Fgf23*^cKO^ mice and cocultured for 21 days with primary osteoblasts that overexpress *Fgf23* (*Fgf23*^TG^) or *Dmp1* (*Dmp1*^TG^) or with isogenic control primary osteoblasts. As expected, DMP1 protein levels were increased in medium from all *Dmp1*^TG^ cocultures, and FGF23 levels were increased in all *Fgf23*^TG^ cocultures (Figure 10, D and E). Isogenic cocultures showed undetectable levels of DMP1 in *Dmp1*^KO^ and *Dmp1*^KO^ *Fgf23*^cKO^ groups and increased levels of FGF23 in the *Dmp1*^KO^ group only. Despite overexpression of *Dmp1* in *Dmp1*^TG^ cocultures, FGF23 levels remained increased in *Dmp1*^KO^, albeit at lower levels than in isogenic cocultures. DMP1 levels were lower in *Fgf23*^TG^ than in isogenic cocultures in the WT and *Fgf23*^cKO^ groups, consistent with effects of FGF23 on osteoblast differentiation. Importantly, isogenic and *Dmp1*^TG^ cocultures showed reduced ALP staining in the *Dmp1*^KO^ group compared with all other groups (Figure 10, F and G). However, ALP staining was reduced in all groups in *Fgf23*^TG^ cocultures, supporting FGF23-induced inhibition of osteoblast differentiation. In addition, and consistent with the stimulatory effects of DMP1 on mineralization, isogenic and *Fgf23*^TG^ cocultures showed reduced ARS staining in the *Dmp1*^KO^ and *Dmp1*^KO^ *Fgf23*^cKO^ groups compared with the WT and *Fgf23*^cKO^ groups (Figure 10, H and I), and *Dmp1*^TG^ cocultures showed increased ARS staining in all groups. Together, our data support an inhibitory effect of FGF23 on osteoblast recruitment and differentiation that is independent from the effects of DMP1 deficiency.

## Discussion

The increase in FGF23 levels, and increased FGF23 signaling in the kidney causing increased Pi excretion, are responsible for hypophosphatemia in hereditary hypophosphatemic disorders (3–6). Conventional therapy that consists of oral Pi and vitamin D supplementation has shown limited efficacy in correcting hypophosphatemia or bone defects associated with hypophosphatemic disorders (10, 31) and may lead to complications such as nephrocalcinosis (32). In contrast, the correction of FGF23 excess, either by reducing its expression or by blocking its signaling, has been shown to improve serum Pi levels, bone growth, and mineralization in patients and animal models with XLH (8, 10, 26, 33). The convergence of phenotypes between XLH and ARHR (4, 5, 21, 22) suggests that humans and animals with ARHR might also benefit from FGF23 correction. In the *Dmp1*-null mouse model of ARHR, genetic overexpression of a *Dmp1* transgene fully rescues FGF23 levels, hypophosphatemia, and bone defects (21, 34–36), but studies specifically targeting FGF23 in models of ARHR are scarce. Previous studies have shown that dietary Pi supplementation improves bone growth and mineralization in 3- to 7-week-old *Dmp1*-null mice (5, 37) and that anti-FGF23 antibodies correct hypophosphatemia and bone mineralization in 1- to 4-week-old *Dmp1*-null mice (37, 38). To date, similar studies have not been performed in adult mice, and anti-FGF23 therapy has only been tested in a limited number of patients with ARHR (14). In the present study, we used dietary Pi supplementation and the osteocyte-specific deletion of *Fgf23* to correct hypophosphatemia in order to study the respective contributions of FGF23 excess, hypophosphatemia, and DMP1 deficiency to the bone defects in adult *Dmp1*-null mice. We further used transcriptomic analyses to study the direct effects of FGF23 and DMP1 on osteoblasts.

In hereditary hypophosphatemia, FGF23 is mainly produced by osteocytes and mature osteoblasts (4, 5, 22, 23, 25, 26). In previous studies performed in Hyp and *Dmp1*-null mice, increased serum iFGF23 levels resulted from increased *Fgf23* expression in bone and impaired posttranslational cleavage of FGF23 (21, 39). Using a *Dmp1*-cre recombinase, we achieved a 75% rescue of serum total and intact FGF23 levels in vivo, and a complete rescue of secreted FGF23 in differentiated primary osteoblasts in vitro, demonstrating that the majority of FGF23 produced in ARHR originates from mature osteoblasts and osteocytes. As anticipated, deletion of *Fgf23* did not modify the i/c FGF23 ratio, which was similar between WT and *Fgf23*^cKO^ mice and increased in *Dmp1*^KO^ and *Dmp1*^KO^ *Fgf23*^cKO^ mice. This suggests that posttranslational cleavage of FGF23 is altered in mice with ARHR regardless of the levels of *Fgf23* expression and is likely due to DMP1 deficiency. In addition, at 12 weeks, *Dmp1*^KO^ mice showed a surprising 3-fold increase in 1,25(OH)_2_D levels compared with WT mice. To our knowledge, this is the only available study that reports 1,25(OH)_2_D levels in adult *Dmp1*^KO^ mice. Previous studies showed normal to low levels of 1,25(OH)_2_D in up to 6-week-old *Dmp1*^KO^ compared with age-matched WT (21, 22, 25). However, values measured in WT mice were much higher in younger mice than in adults, and values measured in 6-week-old *Dmp1*^KO^ mice were similar to those found in the present 12-week-old cohort. Therefore, *Dmp1*^KO^ mice might not show a decline in 1,25(OH)_2_D levels that WT mice may experience with age. The genetic deletion of *Fgf23* in *Dmp1*^KO^ mice reduced PTH levels by 50%, and 1,25(OH)_2_D levels were reduced proportionally to PTH levels, suggesting that the increased 1,25(OH)_2_D levels observed in *Dmp1*^KO^ mice are mainly driven by persistent hyperparathyroidism. While we have not measured intestinal Pi absorption in this model, it is possible that the residual increase in 1,25(OH)_2_D contributes to increased intestinal Pi absorption, which in turns contributes to the full correction of the hypophosphatemia despite residual increases in FGF23 and PTH.

In animal models of XLH and ARHR, the global genetic deletion of *Fgf23* completely suppresses FGF23 production, which results in hyperphosphatemia, hypercalcemia, increased vitamin D levels, ectopic calcifications, and early mortality (23–25, 40). This does not occur with the osteoblastic lineage–specific deletion of *Fgf23* in XLH, which only prevents FGF23 excess and thus restores normal FGF23 and Pi levels (26). As a result, further studies in mice and in patients with XLH showed that a titrated FGF23 blockade significantly increases serum Pi to levels outmatching those achieved by conventional Pi and vitamin D supplementation therapies, with substantial benefits to the skeleton (8, 10). Prior studies in *Dmp1*^KO^ mice showed that dietary Pi supplementation initiated before or at the time of weaning improved bone growth and mineralization (5, 38). In contrast, in the present study, we showed that dietary Pi supplementation, initiated at 6 weeks of age, did not rescue hypophosphatemia or the bone phenotype in 12-week-old *Dmp1*-null mice. Indeed, Pi repletion further increased FGF23 and PTH levels, which exacerbated urinary Pi excretion, osteomalacia, and cortical bone expansion, similarly to studies performed in humans. In aggregate, this suggests that Pi repletion might only be beneficial early in life. In vitro, *Dmp1*-null cultures also did not respond to incremental concentrations of bGP like WT or *Fgf23*^cKO^ cultures and showed persistent alterations of osteoblast differentiation and mineralization despite high bGP supply, suggesting that DMP1 deficiency directly, or indirectly via FGF23 excess, affects osteoblast differentiation and activity.

In prior research (37, 38), blockade of FGF23 signaling using anti-FGF23 antibody administration for 28 days in 6-day-old *Dmp1*^KO^ mice fully corrected hypophosphatemia, bone growth, and trabecular bone volume and partially corrected cortical bone mineralization. In the present study, we show that genetic deletion of *Fgf23* restricted to mature osteoblasts and osteocytes in mice with ARHR led to a partial correction of FGF23 levels, which was sufficient to fully correct hypophosphatemia. In contrast with the administration of anti-FGF23 antibodies in younger mice, and despite the full correction of hypophosphatemia, 12-week-old *Dmp1*-null mice showed only a partial rescue of bone growth and mineralization. We and others (5, 38) show that despite hyperparathyroidism, osteoclast number is reduced and active osteoclast surfaces are normal in *Dmp1*^KO^ bones compared with WT, which suggests that low bone volume in *Dmp1*^KO^ mice is not caused by increased bone resorption and that PTH plays a minimal role in the *Dmp1*^KO^ bone phenotype. Therefore, correction of hypophosphatemia in the face of residual FGF23 elevations is insufficient to correct the bone phenotype associated with ARHR in the long term and the residual bone phenotype might be due to persistent FGF23 elevations and/or DMP1 deficiency. Consistent with this hypothesis, a previous study in the Hyp mouse model of XLH (26) used a Col2.3-cre to delete *Fgf23* in osteoblasts and achieved a similar correction of hypophosphatemia but complete corrections of FGF23 levels, bone growth, and bone morphology. To date, the direct contribution of FGF23 excess to the bone defects associated with hereditary hypophosphatemic disorders is unclear. Studies performed in different models have suggested that FGF23 excess might directly impact osteoblastogenesis (41, 42), reduce osteoblast differentiation (43), suppress alkaline phosphatase (44), and induce the proliferation of the osteoprogenitors at the expense of terminally differentiated cells (42). However, a direct contribution of FGF23 to the osteoblast differentiation and maturation defects in hypophosphatemic rickets has never been shown. Using primary osteoblast cultures and transcriptomics, we identified a Pi-independent role of FGF23 activating FGFR, ERK, and PI3K signaling in *Dmp1*-null osteoprogenitors that results in altered osteoblast differentiation. This was supported by secondary analyses showing that short- and long-term treatment of cultured osteoblasts with FGF23 also led to altered differentiation regardless of their genotype. However, these effects did not dramatically impact mineralization, which appears to remain a distinct effect of DMP1 deficiency. Therefore, we demonstrate that FGF23 excess directly contributes to altered osteoblast differentiation in ARHR and this is specifically rescued by genetic deletion of *Fgf23* in the osteoblast lineage.

While the role of DMP1 as a pro-mineralization factor is well documented (20, 45, 46), its direct contribution in the pathogenesis of impaired mineralization in ARHR is less clear, as most effects are attributed to failure of DMP1 to suppress FGF23 and FGF23-induced hypophosphatemia. In the present study, although genetic deletion of *Fgf23* restored osteoblast differentiation, it did not correct the impaired mineralization observed in *Dmp1*-null osteoblast cultures, suggesting that DMP1 deficiency directly contributes to the mineralization defect despite adequate Pi supply and normal FGF23 production. Interestingly, we show that DMP1 deficiency distinctly results in the reduced expression of genes involved in AMPK and HNF4α signaling. While the role of DMP1 on AMPK signaling needs further investigation, a previous study showed that HNF4α signaling is a central hub linked to FGF23 excess in both Hyp and *Dmp1*^KO^ mice (22). In addition, we recently showed that HNF4α is a novel transcription factor involved in osteogenesis (47), and we believe this is the first study to report common gene targets between DMP1 and HNF4α. Using scRNA-Seq, we further show that DMP1 deficiency directly affects terminally differentiated cells (mature osteoblasts and osteocytes), inhibiting the expression of genes involved in mineral apposition and senescence. This was supported by secondary in vitro analyses showing that delivery of exogenous DMP1 corrected the mineralization defect observed in both *Dmp1*^KO^ and *Dmp1*^KO^ *Fgf23*^cKO^ osteoblasts. Therefore, we demonstrate that, independently of Pi availability and FGF23 production, DMP1 deficiency directly contributes to impaired mineralization in ARHR.

In aggregate, our study suggests that in addition to hypophosphatemia, FGF23 excess and DMP1 deficiency directly control osteoblast differentiation and mineralization in ARHR pathogenesis. While this study supports potential beneficial effects of the use of FGF23 blockade in ARHR disorders, we also show that the full correction of hypophosphatemia is insufficient to fully correct bone growth and mineralization. Our results demonstrate that independently of Pi, residual FGF23 excess directly alters osteoprogenitors’ differentiation, and DMP1 deficiency constitutes an important barrier to adequate bone mineralization in ARHR. Therefore, our data support a potential therapeutic advantage of combined DMP1 repletion and FGF23 blockade to effectively correct ARHR-associated mineral and bone disorders.

## Methods

### Animal studies.

C57BL/6J *Dmp1* global knockout mice (Dmp1^tm1Mis^) were provided by Shanxi Medical University, Taiyuan, China (48). In addition, C57BL/6J mice with an osteocyte-specific deletion of *Fgf23*, i.e., harboring a floxed first exon of the *Fgf23* gene and expressing a transgene containing a Cre recombinase sequence downstream of the *Dmp1* promoter [B6N.FVB-Tg(*Dmp1*-cre)1Jqfe/BwdJ] (*Fgf23^fl/fl^*/*Dmp1-cre^+^*) (49, 50), were provided by University of California, San Francisco, San Francisco, California, USA. We crossed *Fgf23^fl/fl^* *Dmp1-cre^+^* with *Dmp1* heterozygotes (*Dmp1^+/–^*) to generate *Dmp1^+/–^* *Fgf23*^fl/+^ *Dmp1-cre*^(+^^and^^–)^ mice that we further crossed to obtain *Dmp1^+/–^* *Fgf23^fl/fl^* *Dmp1-cre*^(+^^and^^–)^ and *Dmp1^+/–^* *Fgf23^+/+^* *Dmp1-cre*^(+^^and^^–)^ mice. *Dmp1^+/–^* *Fgf23^fl/fl^* *Dmp1-cre*^(+^^and^^–)^ were crossed in a third step to generate experimental WT (*Dmp1^+/+^* *Fgf23^fl/fl^* *Dmp1-cre^–^*), *Fgf23*^Dmp1-cKO^ (*Dmp1^+/+^* *Fgf23^fl/fl^* *Dmp1-cre^+^*), *Dmp1*^KO^ (*Dmp1*^–/–^ *Fgf23^fl/fl^* *Dmp1-cre^–^*), and *Dmp1*^KO^ *Fgf23*^Dmp1-cKO^ (*Dmp1*^–/–^ *Fgf23^fl/fl^* *Dmp1-cre^+^*) littermate mice. *Dmp1^+/–^* *Fgf23^+/+^* *Dmp1-cre*^(+^^and^^–)^ were also crossed to generate experimental WT (*Dmp1^+/+^* *Fgf23^+/+^* *Dmp1-cre^+^*) and *Dmp1*^KO^ (*Dmp1*^–/–^ *Fgf23^+/+^* *Dmp1-cre^+^*) littermates that were homozygotes for the WT *Fgf23* allele. WT and *Dmp1*^KO^ mice were fed either a standard control diet containing 0.7% Pi (NP) or a diet containing 2% Pi (HP) from birth and from 6 weeks of age until 12 weeks of age. Additional WT and *Dmp1*^KO^ mice originated from breeding pairs that were maintained on the HP diet and were kept on HP diet after birth until 12 weeks of age (in utero group). We harvested samples of 12-week-old male littermates and recorded body weight on the day prior to dissection. Each mouse was genotyped twice, once at weaning and after sacrifice, using REDExtract-N-Amp Tissue PCR Kit (MilliporeSigma). We verified DNA recombination of the *Fgf23* allele in mature osteoblasts and osteocytes in mice of each genotype. We isolated bone cells from cortical bone after 12 consecutive extractions, alternating EDTA, trypsin, and collagenase treatments. We filtered the cell suspensions, then sorted 1.0 × 10^5^ mature osteoblasts and osteocytes per sample by FACS, removing matrix debris, nonsingle cells, dead cells, cells larger than 40 μm, and cells with large side-scatter measures. We isolated genomic DNA and amplified the targeted *Fgf23* exon on the sorted cells using the Extract-N-Amp Tissue PCR Kit (MilliporeSigma) and appropriate primer sequences.

### Biochemistry assays.

We collected urine samples after overnight fasting in a metabolic cage and serum samples by intracardiac exsanguination. In all mice, we measured serum iFGF23 levels using a murine iFGF23 ELISA that measures the intact bioactive protein exclusively and total FGF23 (cFGF23) using the cFGF23 ELISA that recognizes the full-length protein and its C-terminal cleavage fragments (both from Quidel). We measured serum PTH using a mouse intact ELISA (Quidel), serum 1,25(OH)_2_D by immunoassay (Immunodiagnostic Systems), and serum and urine Pi and calcium using colorimetric assays (Pointe Scientific). We also measured cFGF23 (Quidel) and total DMP1 using a mouse ELISA that recognizes the full-length and the C-terminal–cleaved DMP1 proteins (Abcam) in cell culture media collected on the last day of culture.

### High-resolution 3D μCT.

We scanned ethanol-fixed whole femurs with high-resolution microtomography (μCT50; Scanco Medical) at 2 μm isotropic voxel size, energy level of 70 keV, and intensity of 57 μA. The trabecular bone structure was analyzed within 1.2 mm of the secondary spongiosa of the distal femur underneath the growth plate. The cortical bone structure was analyzed within 1 mm at the midshaft of each femur. All grayscale images were segmented using a fixed Gaussian filter and threshold for all data. Representative segmented images were generated for the trabecular bone and color scale heatmaps of local density for the cortical bone, as previously shown. Finally, cortical pore density and maximum diameter were analyzed in 1.2 mm regions of the diaphysis, and we generated representative color scale heatmaps of cortical pores maximum diameters. Whole mice were scanned at a resolution of 10 μm isotropic voxel size.

### Histology and acid-etched SEM.

We injected mice with ARS at 7 and 2 days prior to harvest for intravital staining of active mineralization fronts (21, 51). We measured femur and tail lengths using a slide caliper to evaluate bone growth (21, 22, 51). We fixed and dehydrated the femur samples in ethanol, and we embedded them in methylmetacrylate (MMA) at 4°C. For SEM analyses, we acid-etched the polished surface of the MMA block with 37% phosphoric acid for 2–10 seconds, washed twice with water followed by 5% sodium hypochlorite for 5 minutes, and washed again in water. We coated the air-dried samples with gold and palladium, then examined by FEI/Philips XL30 Field emission environmental SEM according to previously described protocol (5). For histology analyses, we cut nonserial 5 μm MMA slices (Leica Microsystems) and captured bright-field and fluorescence microscopy images (Leica Microsystems). We analyzed unstained longitudinal femoral sections, modified trichrome Goldner–stained sections, and TRAcP activity–stained sections according to previously described methods (52).

### Cell cultures.

We prepared bone marrow stromal cells from 12-week-old WT, *Fgf23*^cKO^, *Dmp1*^KO^, and *Dmp1*^KO^ *Fgf23*^cKO^ mice according to a previously described protocol (19). We plated 10 × 10^4^ cells per well and cultured for 14 and 21 days in osteoblast-differentiating medium (α-minimal essential medium, 10% fetal bovine serum, 10 U/mL penicillin, 100 μg/mL streptomycin, 3/7/10 mmol/L bGP, and 50 μg/mL ascorbic acid) prior to staining and collection. For coculture experiments, cells were plated at the same density on plates or sterile 0.4 μm pore size 12-well format cell culture inserts (Corning) and cultured for 21 days in osteoblast-differentiating medium. We stained with ALP and ARS and quantified staining following previously described protocols (19). Prior to fixation, we incubated the cells for 1 hour in 1 mL culture medium containing 10% Alamar blue (Invitrogen). We assessed the number of cells by plotting the absorbance of incubated cells against a standard curve of MC3T3-E1 cultures. We cultured MC3T3-E1 (Subclone 4, CRL-2593, ATCC) osteoblasts for 21 days in osteoblast differentiation medium of similar composition containing 10 mmol/L bGP and 0/25/50 ng/mL of mouse recombinant FGF23 (R&D Systems).

### Real-time PCR.

We isolated total RNA from kidney and bone tissues and from MC3T3-E1 cultures using TRI reagent and synthesized first-stand cDNA (iScript cDNA Synthesis Kit, Bio-Rad Laboratories). We used the iCycler iQ real-time PCR detection system with iQ SYBR Green supermix (Bio-Rad Laboratories) for real-time quantitative PCR analysis. The threshold of detection of each gene expression was set at optimal reaction efficiency. The expression was plotted against a standard dilution curve of relative concentration, normalized to *Rpl19* expression in the same sample, and expressed as fold-change versus WT.

### Bulk RNA-Seq.

We prepared the total RNA library for each individual sample using the TruSeq Total RNA-Seq Library Preparation Kit (Illumina), then sequenced the bar-coded cDNA libraries for 50 bp paired-end reads using Illumina NovaSeq 6000 to generate 80 million to 90 million paired reads/sample. We mapped the reads from each library to the mouse transcriptome and genome, filtered using StrandNGS software suite (Strand Life Sciences), following Strand alignment and filtering pipelines. We normalized the reads using DESeq, and we used baseline transformation to the median for all samples. We calculated the fold-change and *P* value using moderated 2-tailed *t* test. For all analyses, we used a 2-pass enrichment method, by first computing the list of genes based on uncorrected *P* < 0.1, and we intersected these data sets to determine FGF23- and DMP1-regulated genes. The final list of genes was used as input to calculate differentially expressed genes at *P* < 0.1 using moderated 2-tailed *t* test and Bonferroni-Holm corrections. We used the final data sets for subsequent downstream pathway analyses using the IPA platform (QIAGEN).

### scRNA-Seq.

We isolated and cultured bone marrow stromal cells from adult WT, *Fgf23*^cKO^, *Dmp1*^KO^, and *Dmp1*^KO^ *Fgf23*^cKO^ mice for 21 days in osteoblastic medium. We isolated 3 individual cultures per genotype at day 21 by a single incubation with 1% trypsin for 10 minutes, to minimize cell stress. We incubated each cell suspension with individual 3′ CellPlex oligos (10x Genomics, catalog PN-1000261) and 7-AAD for cell viability, then sorted them on a BD FACSAria SORP system. We pooled the suspensions by genotype and suspended in PBS 1% BSA solution. We analyzed cell number and viability after sorting using the Nexcelom Cellometer Auto 2000 with the AOPI fluorescent staining method, which resulted in single-cell suspensions with more than 90% viability. We loaded 10,000 viable cells from each cell suspension into the Chromium Controller (10x Genomics; catalog PN-120223) on a Chromium Next GEM Chip G (10x Genomics, catalog PN-1000120) and processed to generate single-cell gel beads in the emulsion (GEM) according to the manufacturer’s protocol. We generated the cDNA and libraries using the Chromium Next GEM Single Cell 3′ Kit v3.1 (10x Genomics; catalog PN-1000268), Dual Index Kit TT Set A (10x Genomics; catalog PN-1000215), and Dual Index Kit NN Set A (for sample multiplexing) (10x Genomics; catalog PN-1000243) according to the manufacturer’s manual. We performed the quality control for the constructed library using the Bioanalyzer High Sensitivity DNA Kit (Agilent Technologies, catalog 5067-4626), and we used Qubit 4.0 Fluorometer (Thermo Fisher Scientific, catalog PN-Q33238) for qualitative and quantitative analysis. We pooled and sequenced the multiplexed libraries on an Illumina NovaSeq 6000 sequencer. We demultiplexed raw sequencing data in base call format (.bcl) using Cell Ranger (10x Genomics), converting the raw data into FASTQ format to generate a gene expression library and a cell multiplexing library per sample. We processed, demultiplexed, and aligned the samples using a 2-pass Cell Ranger multianalysis. First, we reprocessed Cell Ranger output with HTO demux, then reran Cell Ranger multi using the assigned barcodes for each sample, to create 4 replicates per genotype, 3 using the specific sample barcode and a fourth from the untagged nondoublet cells. We imported the resulting matrix files, which summarize the alignment results, into R package Seurat (Satija Lab, New York Genome Center) for further analysis (53). In Seurat (v4.3.0), we transformed each individual sample to a Seurat object. We next merged the data from all 16 samples and used SCT transform to normalize and scale the data regressing out mitochondrial genes [SCTransform(obj, vars.to.regress = “percent.mt”)]. Then, we processed the combined object using the standard Seurat workflow, including principal component analysis, UMAP, and neighboring and clustering analyses using the first 30 principal components, at a resolution of 0.8. We visualized the cell clusters in 2D space by UMAP. All genotypes showed similar clustering and cell populations in separate and overlapping DimPlot (54). To identify cell clusters, we assessed marker genes using FindAllMarkers function of Seurat. We annotated the clusters based on the expression of top marker genes supported by literature search and scMCA (https://github.com/ggjlab/scMCA; commit ID 0ac6b52). We isolated the osteoblastic clusters from the original Seurat object, which we further analyzed. We performed differential gene expression analysis by cluster using a 2-pass enrichment method. First, we selected all differentially expressed genes with unadjusted *P* < 0.1, which we exported to IPA for comparison analyses. We next used the final list of genes resulting from the comparison analyses as input in StrandNGS to calculate differentially expressed genes at *P* < 0.1 using moderated 2-tailed *t* test and Bonferroni-Holm corrections. Genes with a corrected *P* < 0.1 were considered significant and used in IPA for pathway analysis and network design. To perform the pseudotime analysis on the Seurat object, we analyzed the osteoblast cell subset using the R package Monocle 3 (55), then added the pseudotime score to the metadata of the Seurat object.

### Statistics.

Data are presented as mean ± SEM. We used 2-tailed *t* tests or 1-way ANOVA followed by post hoc 2-tailed *t* tests and multiple-testing correction using Bonferroni or Holm-Bonferroni method (56) (Statistica software, Statsoft). Differences were considered statistically significant at *P* < 0.05 or *P* < 0.1 as indicated in the figure legends.

### Study approval.

All animal studies were conducted in accordance with the Northwestern University Institutional Animal Care and Use Committee.

### Data availability.

All data associated with this study are presented in the manuscript and in the Supporting Data Values file. Materials and protocols are available upon request. Next-generation sequencing data were deposited to National Center for Biotechnology Information Gene Expression Omnibus (GEO) as GEO SuperSeries GSE239830 as follows: bulk RNA-Seq data as GSE239825 and scRNA-Seq data as GSE239828.

## Author contributions

GC, DK, VD, and AM designed the experiments. GC, DK, JJT, Xueyan Wang, LMN, SL, JS, and MMC conducted the experiments. GC, DK, JJT, Xueyan Wang, HHT, LMN, SL, JS, JVD, MMC, LH, and Xiaofang Wang acquired and analyzed the data. WC and JQF provided mouse models. GC, DK, VD, and AM wrote the manuscript. All authors edited and approved the final version of the manuscript.

## Supplementary Material

Supplemental data

Supporting data values

## Version 1 11/09/2023

In-Press Preview

## Version 2 12/22/2023

Electronic publication
