# Supplementary material for: FGF23 directly inhibits osteoprogenitor differentiation in Dmp1-knockout mice
Source: JCI Insight. 2023 Dec 22;8(24):e156850. doi: 10.1172/jci.insight.156850 (PMC10807721; doi:10.1172/jci.insight.156850)
Supplement: Supplemental data [file jciinsight-8-156850-s070.pdf]

SUPPLEMENTAL FIGURES

Figure S1

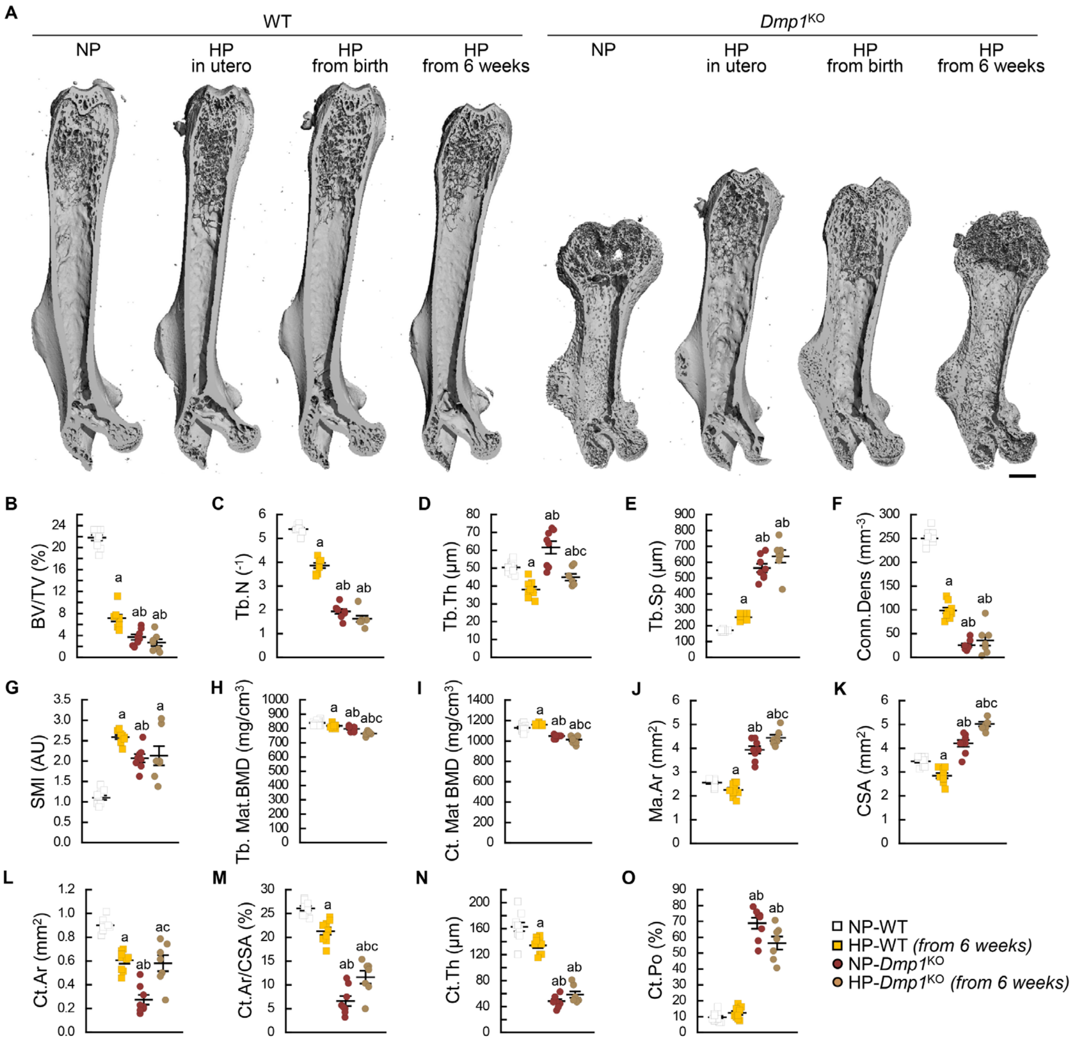

Figure S1: Dietary phosphate supplementation worsens bone microarchitecture in WT and in *Dmp1*^{KO} mice. (**A**) 3D-µCT representation of total femur in sagittal plane (scale bar = 1 mm) in 12 week-old WT and *Dmp1*^{KO} fed a diet containing 0.7% Pi (normal Pi - NP) or 2% Pi (high Pi - HP) in utero, from birth or from 6 weeks. 3D-µCT parameters of (**B-H**) trabecular bone microarchitecture and (**I-O**) cortical bone microarchitecture in 12 week-old WT (n≥9) and *Dmp1*^{KO} (n≥7) fed a diet containing 0.7% Pi (normal Pi - NP) or 2% Pi (high Pi - HP) from 6 to 12 weeks of age. Values are expressed as mean ± SEM; $p<0.05$ vs. [a] NP-WT, [b] HP-WT, [c] NP-*Dmp1*^{KO}. Statistical tests were performed using ANOVA test followed by post-hoc t tests and multiple testing correction using Holm-Bonferroni method.

Abbreviations: bone volume to tissue volume ratio (BV/TV), trabecular number (Tb.N), thickness (Tb.Th), trabecular separation (Tb.Sp), connectivity density (Conn.Dens), structural model index (SMI), material bone mineral density (mat BMD), marrow area (Ma.Ar), cross sectional area (CSA), cortical area (Ct.Ar), Ct.Ar to CSA ratio (Ct.Ar/CSA), cortical thickness (Ct.Th), and cortical porosity (Ct.Po).

Figure S2

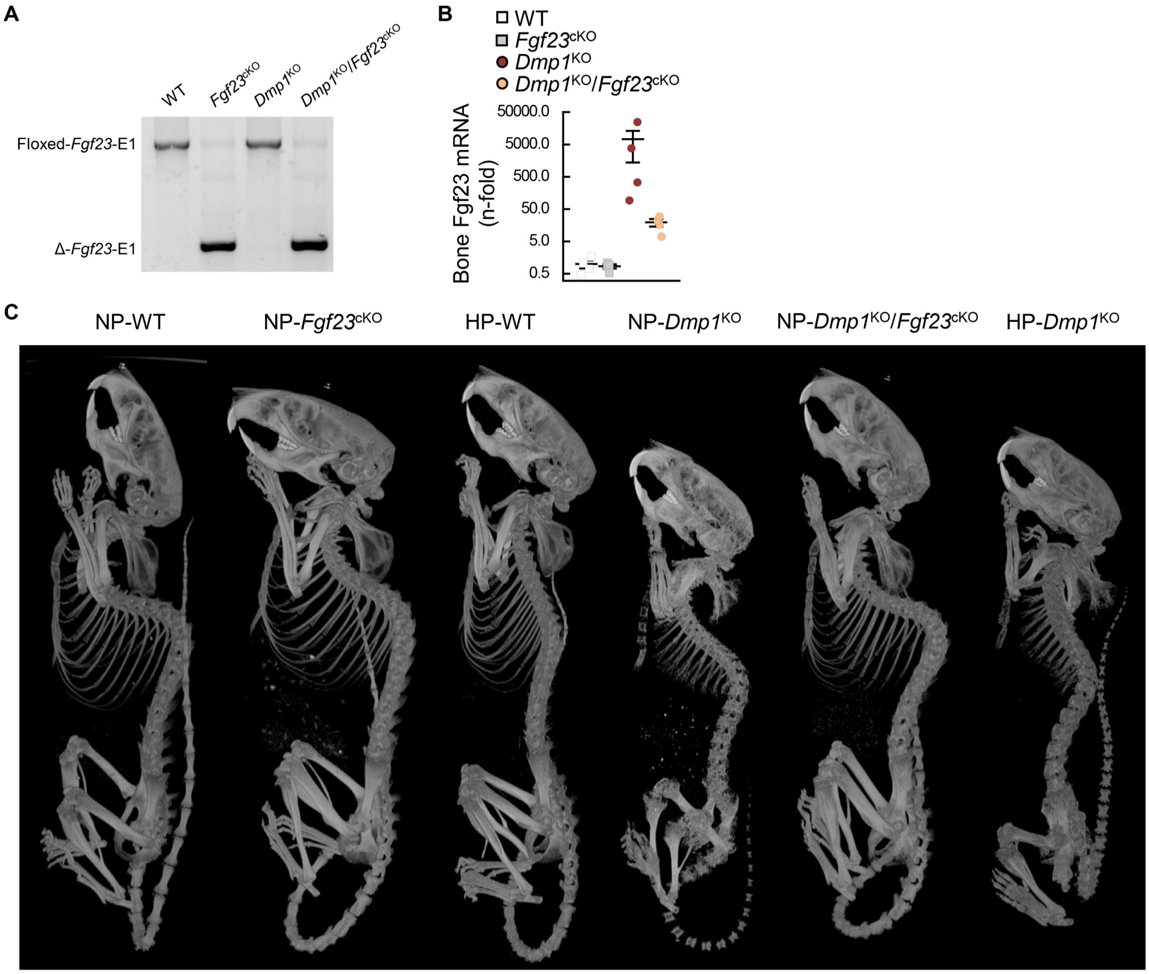

Figure S2: Comparative analysis between the bone-specific deletion of *Fgf23* and dietary Pi supplementation in WT and *Dmp1*^{KO} mice. (**A**) DNA amplification of the floxed *Fgf23* allele in WT and *Dmp1*^{KO} mice with and without expression of the Dmp1 cre recombinase showing the non-recombined floxed exon 1 of *Fgf23* in *Dmp1*$^{+/+}$;*Fgf23*$^{flox/flox}$;*Dmp1*-cre^{-} (WT) and *Dmp1*$^{-/-}$;*Fgf23*$^{flox/flox}$;*Dmp1*-cre^{-} (*Dmp1*^{KO}) mice and the recombined exon 1 of *Fgf23* in *Dmp1*$^{+/+}$;*Fgf23*$^{flox/flox}$;*Dmp1*-cre^{+} (*Fgf23*^{cKO}) and *Dmp1*$^{-/-}$;*Fgf23*$^{flox/flox}$;*Dmp1*-cre^{+} (*Dmp1*^{KO}/*Fgf23*^{cKO}) mice. (**B**) Bone *Fgf23* mRNA expression in 12-week-old WT (n≥4), *Fgf23*$^{Dmp1\text{-cKO}}$ (n≥4), *Dmp1*^{KO} (n≥4) and *Dmp1*^{KO}/*Fgf23*$^{Dmp1\text{-cKO}}$ (n≥4) mice. (**C**) 3D-µCT whole body scan of 12-week-old WT, *Fgf23*$^{Dmp1\text{-cKO}}$, *Dmp1*^{KO} and *Dmp1*^{KO}/*Fgf23*$^{Dmp1\text{-cKO}}$ fed a diet containing 0.7% Pi (normal Pi - NP) or 2% Pi (high Pi - HP) from 6 to 12 weeks of age. Values are expressed as mean ± SEM; $p<0.05$ vs. [a] WT, [b] *Fgf23*^{cKO}. Statistical tests were performed using ANOVA test followed by post-hoc t tests and multiple testing correction using Holm-Bonferroni method.

Figure S3

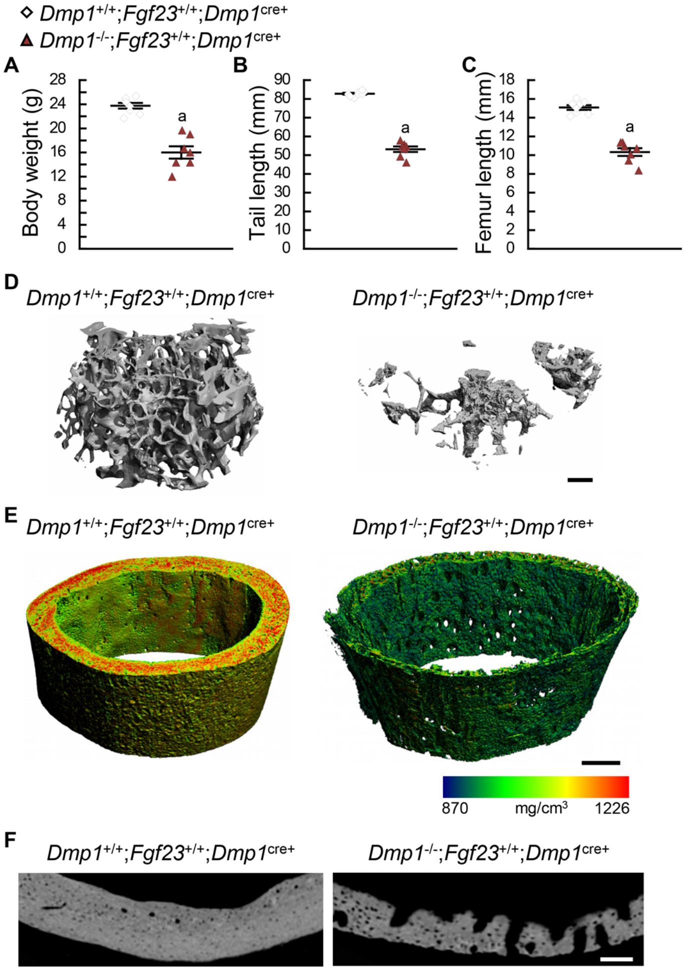

Figure S3: Dmp1-cre does not affect mice homozygous for the wild-type *Fgf23* allele. (**A**) Body weight, (**B**) tail length, (**C**) femur length, 3D-µCT scan reconstruction of (**D**) distal femur trabecular metaphysis (scale bar = 250 µm) and (**E**) midshaft femur cortical diaphysis (scale bar = 250 µm), and (**F**) 2D-µCT analysis of cortical bone porosity (scale bar = 100 µm) in 12 week-old *Dmp1*$^{+/+}$;*Fgf23*$^{+/+}$;*Dmp1*-cre^{+} (WT, n=8) and *Dmp1*$^{-/-}$;*Fgf23*$^{+/+}$;*Dmp1*-cre^{+} (*Dmp1*^{KO}, n=7) mice mice that expressed the Dmp1-cre recombinase and were homozygous for the wild-type Fgf23 allele. Values are expressed as mean ± SEM; p<0.05 vs. [a] WT. Statistical tests were performed using unpaired student's t-tests.

Figure S4

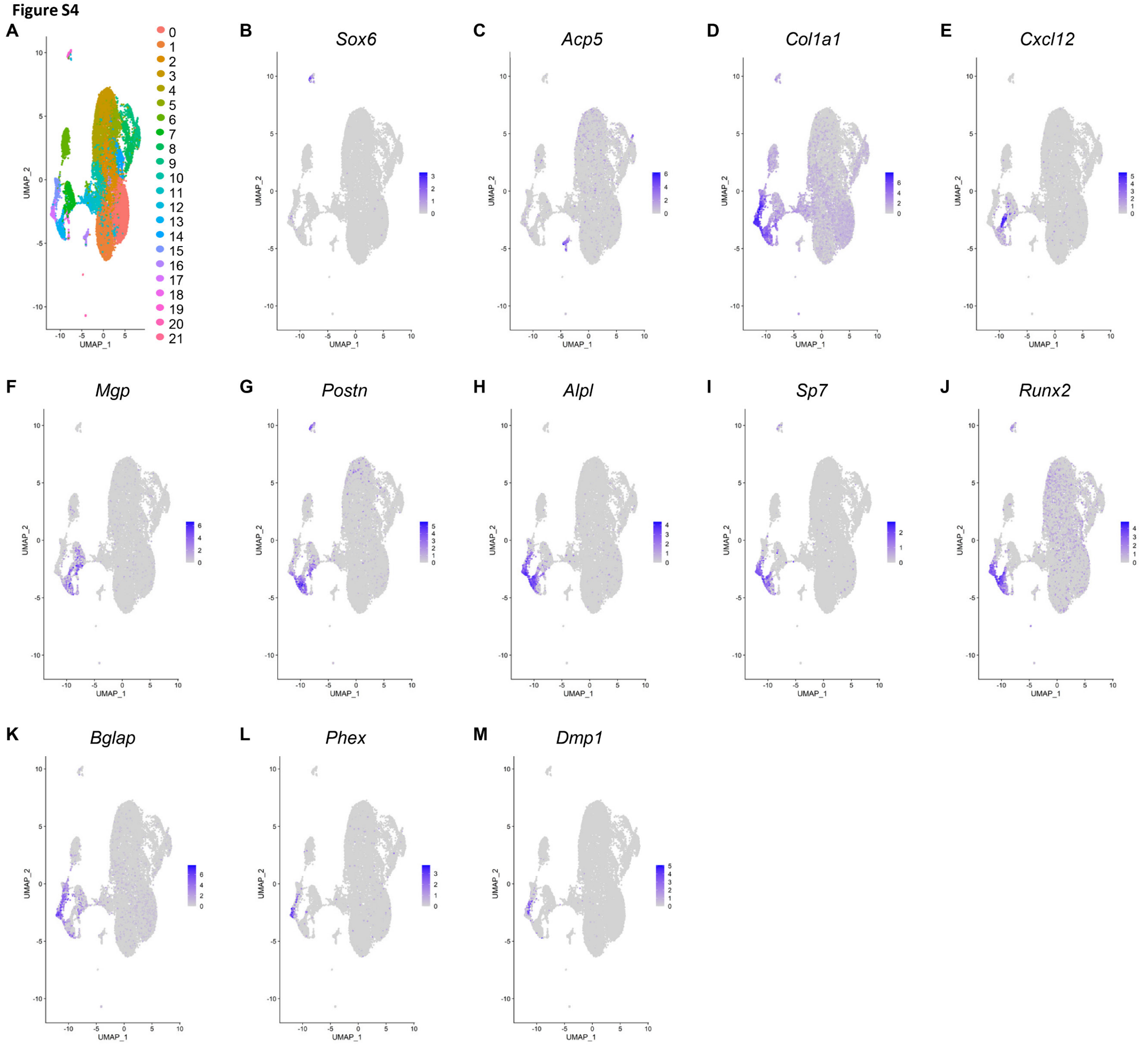

Figure S4: Expression of osteoblast differentiation markers at the single cell level. Single cell RNA sequencing analysis was performed on bone marrow stromal cells isolated from 12-week-old WT (n=3), *Fgf23*$^{Dmp1\text{-cKO}}$ (n=3), *Dmp1*^{KO} (n=3) and *Dmp1*^{KO}/*Fgf23*$^{Dmp1\text{-cKO}}$ (n=3) mice and cultured for 21 days in osteoblast differentiation medium containing 10 mM of beta-glycerophosphate. (**A**) Uniform manifold approximation and projection (UMAP) plot shows 21 different cell clusters. (**B-M**) Feature plots representing the distribution of the expression of osteoblast specific markers in all clusters.

Figure S5

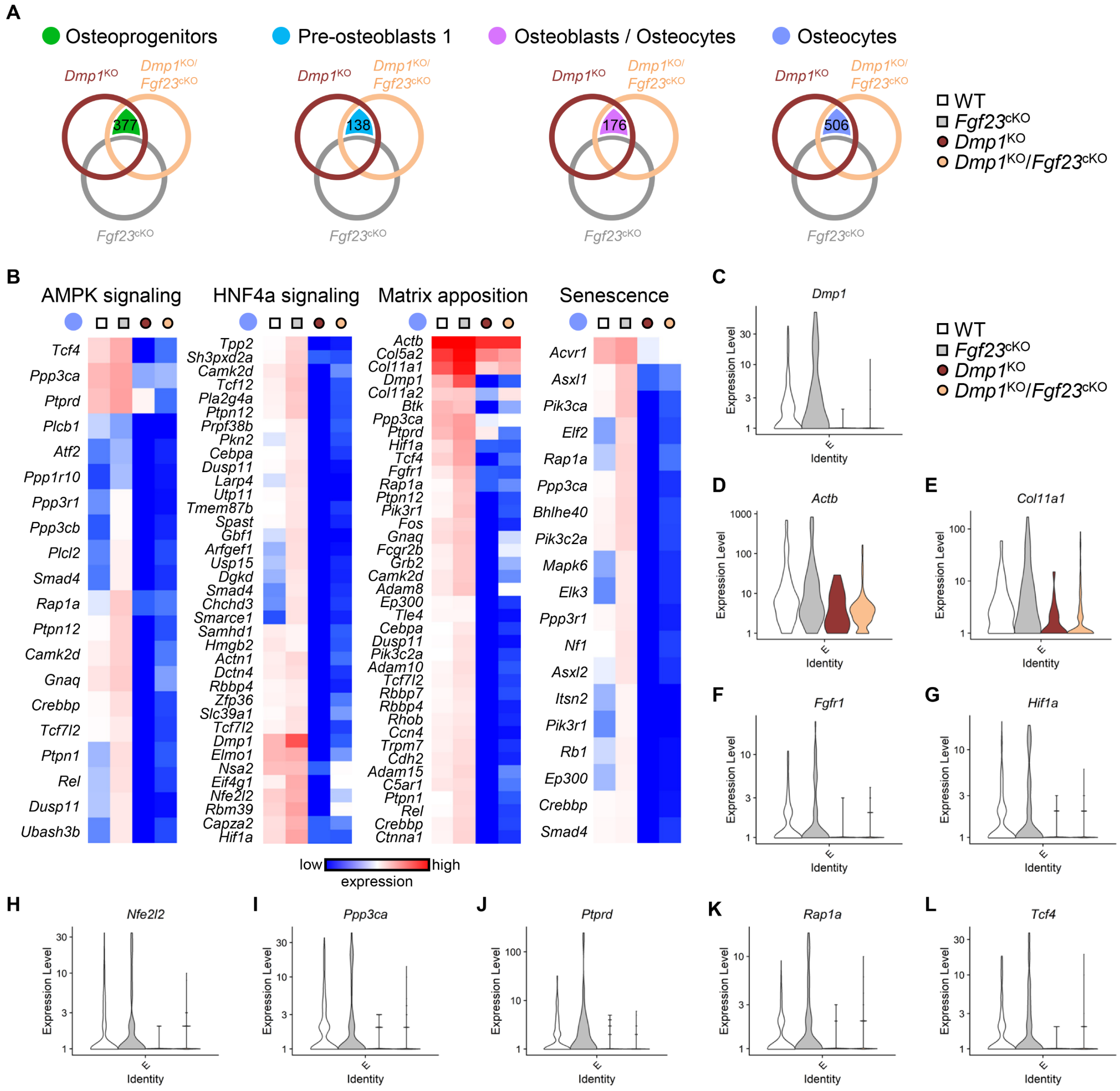

Figure S5: DMP1 deficiency impairs mineralization via inhibition of the AMPK and HNF4α signaling pathways in osteocytes. Single cell RNA sequencing analysis was performed on bone marrow stromal cells isolated from 12-week-old WT (n=3), *Fgf23*$^{Dmp1\text{-cKO}}$ (n=3), *Dmp1*^{KO} (n=3) and *Dmp1*^{KO}/*Fgf23*$^{Dmp1\text{-cKO}}$ (n=3) mice and cultured for 21 days in osteoblast differentiation medium containing 10 mM of beta-glycerophosphate. (**A**) Venn diagram identifies genes showing altered expression in *Dmp1*^{KO} and in *Dmp1*^{KO}/*Fgf23*^{cKO} osteoblasts (*colored area*) in each cluster of differentiation. (**B**) Heatmaps represent the expression of genes identified in (A) in the osteocytes cluster (*lavender dot*) and used in Ingenuity Pathway Analysis (IPA) to define the most represented canonical pathways regulated by DMP1. (**C-L**) Violin plots showing the expression of representative target genes in *Dmp1*^{KO} and *Dmp1*^{KO}/*Fgf23*^{cKO} osteocytes. Statistical tests were performed using Mann-Whitney's U test and corrected by the False Discovery Rate ($p<0.1$).

SUPPLEMENTAL TABLES

Table S1: 3D microtomography analysis of cortical bone moments of inertia from 12-week-old WT, *Fgf23*^{cKO}, *Dmp1*^{KO} and *Dmp1*^{KO}/*Fgf23*^{cKO} mice.

	**WT**	***Fgf23*^{cKO}**	***Dmp1*^{KO}**	***Dmp1*^{KO}/*Fgf23*^{cKO}**
**pMOI (mm^4)**	0.40 ± 0.02	0.53 ± 0.03 [a]	0.20 ± 0.04 [ab]	0.48 ± 0.03 [c]
**Imin (mm^4)**	0.13 ± 0.01	0.17 ± 0.01	0.06 ± 0.01 [ab]	0.15 ± 0.01 [c]
**Imax (mm^4)**	0.27 ± 0.01	0.36 ±0.02 [a]	0.14 ± 0.03 [ab]	0.32 ± 0.02 [c]

Values are represented as mean ± SEM; n=5 (WT, *Fgf23*^{cKO}, *Dmp1*^{KO}/*Fgf23*^{cKO}); n=6 (*Dmp1*^{KO}); Corrected $p<0.05$ vs. [a] WT, [b] *Fgf23*^{cKO}, [c] *Dmp1*^{KO}. Statistical tests were performed using ANOVA test followed by post-hoc t-tests and multiple testing correction using Holm-Bonferroni method.

Abbreviations: pMOI: polar moment of inertia; Imin: minimum moment of inertia; Imax: maximum moment of inertia.

Table S2: Biochemistry analysis of phosphate metabolism markers in 12-week-old WT and *Dmp1*^{KO} mice harboring the expression of the Cre recombinase under the control of Dmp1 promoter.

	Dmp1$^{+/+}$**;*Fgf23***$^{+/+}$**;*Dmp1***$^{cre+}$	***Dmp1***$^{-/-}$**;*Fgf23***$^{+/+}$**;*Dmp1***$^{cre+}$
**Serum cFGF23 (pg/mL)**	354.1 ± 20.2	3396.5 ± 372.5 [a]
**Serum iFGF23 (pg/mL)**	190.3 ± 11.5	3024.4 ± 206.5 [a]
**Serum PTH (pg/mL)**	257.1 ± 29.6	948.3 ± 146.3 [a]
**Serum Pi (mg/dL)**	7.9 ± 0.3	5.5 ± 0.4 [a]
**FePi (%)**	2.8 ± 0.6	4.8 ± 0.3 [a]

Values are represented as mean ± SEM; n≥4; [a] $p<0.05$ vs. *Dmp1*$^{+/+}$;*Fgf23*$^{+/+}$;*Dmp1*$^{cre+}$. Statistical tests were performed using unpaired student's t tests.

Table S3: 3D microtomography parameters of trabecular and cortical bone microarchitecture in 12-week-old WT and *Dmp1*^{KO} mice harboring the expression of the Cre recombinase under the control of Dmp1 promoter.

	Dmp1$^{+/+}$**;*Fgf23***$^{+/+}$**;*Dmp1***$^{cre+}$	***Dmp1***$^{-/-}$**;*Fgf23***$^{+/+}$**;*Dmp1***$^{cre+}$
Trabecular bone		
**BV/TV (%)**	15.3 ± 1.6	1.8 ± 0.4 [a]
**Tb.N (mm**$^{-1}$**)**	5.0 ± 0.2	1.7 ± 0.2 [a]
**Tb.Th (µm)**	43.7 ± 0.7	62.9 ± 6.9 [a]
**Tb.Sp (µm)**	185.7 ± 6.9	639.4 ± 41.0 [a]
**Conn.Dens (mm**$^{-3}$**)**	215.2 ± 25.2	7.4 ± 2.4 [a]
**SMI**	1.8 ± 0.2	2.7 ± 0.2 [a]
Cortical bone		
**Mat BMD (mg/cm**^{3}**)**	1135.4 ± 11.4	1044.5 ± 5.7 [a]
**Ma.Ar (mm**^{2}**)**	2.2 ± 0.1	3.8 ± 0.3 [a]
**CSA (mm**^{2}**)**	3.0 ± 0.2	4.1 ± 0.4 [a]
**Ct.Ar (mm**^{2}**)**	0.76 ± 0.04	0.25 ± 0.06 [a]
**Ct.Ar/CSA (%)**	25.9 ± 1.4	5.8 ± 0.9 [a]
**Ct.Th (µm)**	152.7 ± 4.4	47.8 ± 3.8 [a]
**Ct.Po (%)**	8.2 ± 0.5	73.2 ± 2.6 [a]

Values are represented as mean ± SEM; n=5 (*Dmp1*$^{+/+}$;*Fgf23*$^{+/+}$;*Dmp1*$^{cre+}$); n=6 (*Dmp1*$^{-/-}$;*Fgf23*$^{+/+}$;*Dmp1*$^{cre+}$); [a] $p<0.05$ vs. *Dmp1*$^{+/+}$;*Fgf23*$^{+/+}$;*Dmp1*$^{cre+}$. Statistical tests were performed using unpaired student's t tests.

Abbreviations: BV/TV: bone volume to tissue volume ratio; Tb.: trabecular; N: number; Th: thickness; Sp: separation; Conn.Dens: connectivity density; SMI: structure model index; Mat BMD: material bone mineral density; Ma.Ar: marrow area; CSA: cross sectional area; Ct: cortical; Po: porosity.

Table S4: 2D histomorphometry analysis of trabecular bone resorption in distal femur secondary spongiosa from 12-week-old WT, *Fgf23*^{cKO}, *Dmp1*^{KO} and *Dmp1*^{KO}/*Fgf23*^{cKO} mice.

	**WT**	***Fgf23*^{cKO}**	***Dmp1*^{KO}**	***Dmp1*^{KO}/*Fgf23*^{cKO}**
**N.Oc/B.Ar (mm^{-2})**	505.5 ± 37.0	324.3 ± 34.8 ^{a}	45.5 ± 24.6 ^{ab}	112.9 ± 21.4 ^{ab}
**N.Oc/B.Pm (mm^{-1})**	7.6 ± 0.4	4.8 ± 0.5 ^{a}	1.4 ± 0.8 ^{ab}	2.9 ± 0.4 ^{ab}
**Oc.S/BS (%)**	16.0 ± 2.1	12.2 ± 1.3	5.0 ± 2.8	8.7 ± 1.5

Values are represented as mean ± SEM; n=4 (WT, *Fgf23*^{cKO}, *Dmp1*^{KO}/*Fgf23*^{cKO}); n=3 (*Dmp1*^{KO}); $p<0.05$ vs. ^{a} WT, ^{b} *Fgf23*^{cKO}. Statistical tests were performed using ANOVA test followed by post-hoc t-tests and multiple testing correction using Holm-Bonferroni method.

Abbreviations: N.Oc: number of osteoclasts; B.Ar: bone area; B.Pm: bone perimeter; Oc.S: osteoclast surface; BS: bone surface.
